# Implications of Network Topology on Stability

**DOI:** 10.1371/journal.pone.0122150

**Published:** 2015-03-31

**Authors:** Ali Kinkhabwala

**Affiliations:** 1 Department of Systemic Cell Biology, Max Planck Institute of Molecular Physiology, Dortmund, Germany; Manchester University, UNITED KINGDOM

## Abstract

In analogy to chemical reaction networks, I demonstrate the utility of expressing the governing equations of an arbitrary dynamical system (interaction network) as sums of real functions (generalized *reactions*) multiplied by real scalars (generalized *stoichiometries*) for analysis of its stability. The reaction stoichiometries and first derivatives define the network’s “influence topology”, a signed directed bipartite graph. Parameter reduction of the influence topology permits simplified expression of the principal minors (sums of products of non-overlapping bipartite cycles) and Hurwitz determinants (sums of products of the principal minors or the bipartite cycles directly) for assessing the network’s steady state stability. Visualization of the Hurwitz determinants over the reduced parameters defines the network’s stability phase space, delimiting the range of its dynamics (specifically, the possible numbers of unstable roots at each steady state solution). Any further explicit algebraic specification of the network will project onto this stability phase space. Stability analysis via this hierarchical approach is demonstrated on classical networks from multiple fields.

## Introduction

In this manuscript, I introduce a hierarchical approach (topology-then-algebra) to the bifurcation analysis of arbitrary dynamical systems (those definable by an autonomous set of ordinary differential equations, ODEs). Specifically, I show how a network’s bipartite “influence topology”—elsewhere referred to as its Directed Species Reaction (DSR) graph, first introduced by Banaji & Craciun in 2009 [1, 2]—provides a tool for systematic exploration of the fundamental Routh-Hurwitz conditions for local steady state stability [3–11].

The outline of my manuscript is as follows. I first introduce a topological interpretation of the Routh-Hurwitz conditions as products of non-overlapping and overlapping *unipartite* cycles in the network (§1). I then establish that these expressions can be immediately reinterpreted in terms of the *bipartite* cycles of the influence topology (§2). The graph of a particular network’s influence topology will contain *J* Jacobian (derivatives of each reaction with respect to each relevant species) and *S* stoichiometric edges, giving at most *J*+*S* parameters that determine the Routh-Hurwitz conditions. Reductions of these *J*+*S* parameters to often far fewer parameters is demonstrated through stoichiometric scaling, “cycle compaction”, and temporal scaling. Such reductions will be referred to as “parameter reductions” in order to avoid any confusion that might stem from use of the term “dimensional reductions”, which more conventionally pertains to reductions in the dimensionality of the original set of governing ODEs for the network. Stoichiometric reduction refers to the setting to unity of a single stoichiometric edge at each reaction node, amounting to a trivial rescaling of the expressions defining each reaction. What I refer to as “cycle compaction” is less trivial. Cycles in the bipartite graph of the influence topology are defined as products of sequential (alternating) Jacobian and stoichiometric edges. As already mentioned above, the Routh-Hurwitz conditions depend only on these bipartite cycles, which are generally highly overlapping. For each particular type of overlap between some exact number of bipartite cycles, the product of the edges that define the overlap can be reduced to a single cycle compaction parameter. Instead of its full product of Jacobian and stoichiometric edges, any bipartite cycle in the network can be defined in terms of the relevant cycle compaction parameters. Following stoichiometric scaling and cycle compaction, a single temporal parameter (either a Jacobian edge itself or a cycle compaction parameter containing at least one Jacobian edge) can be scaled to unity, amounting to a trivial rescaling of the time axis. These three forms of parameter reduction allow expression of the Routh-Hurwitz conditions using often far fewer parameters. The negativity of individual Hurwitz determinants (indicating instability) can be displayed over the entire domain of these reduced parameters allowing visualization of the network’s “stability phase space”. This permits rapid evaluation of the particular regions of stability and instability for the network (§3). A given stability phase space will be dissected into distinct instability zones over which one or more Hurwitz determinants are negative, with the Routh array allowing determination of the exact number of unstable eigenvalues in each zone or zone overlap. Significantly, the potential for a Hopf bifurcation can be immediately assessed through examination of the stability phase space for the possibility of a transition from a stable zone to one in which the last and penultimate Hurwitz determinants simultaneously go negative. Several important networks are examined using this overall approach, including networks comprised of a single *n*-cycle (§4) and classical networks studied in various fields (§5). A starting point for the analysis of more general networks is introduced involving the upstream/downstream partitioning of a given network’s influence topology (§6), with isolated sets of overlapping cycles (or, in graph theory parlance, “strongly connected components”) used to demarcate the distinct levels in the graph. Based on the utility of this upstream/downstream partitioning, the notion of a *fundamental* set of influence topologies constructed solely from overlapping cycles is presented with important degeneracies within this set identified (§7). In the final Discussion section, a broader perspective is explored within the context of multiple open questions.

## Results

### 1 Expression of the Routh-Hurwitz Conditions for a Network in Terms of its Unipartite Cycles

In this section, it is shown that the Routh-Hurwitz conditions for a given dynamical system (interaction network) can be written solely in terms of products of its unipartite (species-only) cycles. Consider the following arbitrary system of autonomous first-order ODEs:

$$\begin{array}{c} \frac{dx_{j}}{dt}=f_{j}\left(x_{1},\ldots ,x_{n}\right), \end{array}$$ (1)

with *j* ranging from 1 to *n*. The *f*_*j*_(*x*_1_, …, *x*_*n*_) denote real-valued functions of the real variables *x*_*j*_ (species). This general definition encompasses many important interaction networks studied in control theory, biology, chemistry, physics, and electronics [12]. Upon setting all ${\dot{x}}_{j}$ to zero, the one or more steady state solutions of the system can be found through solution of the system of equations: *f*_*j*_(*x*_1_, …, *x*_*n*_) = 0. Perturbation about a particular steady state solution $\left(x_{1}^{s},\ldots ,x_{n}^{s}\right)$ yields to first order:

$$\begin{array}{c} \frac{d\Delta x_{j}}{dt}≃\sum_{i}\Delta x_{i}H_{ij}, \end{array}$$ (2)

with *H*_*ij*_ ≡ (∂*f*_*j*_/∂*x*_*i*_)_*s*_ the “transition rate constants” from *i* to *j* defined at the steady state *s*. In matrix form, this can be written as:

$$\begin{array}{c} \frac{d}{dt}\Delta x≃\Delta x\cdot H, \end{array}$$ (3)

with **Δx** a row vector. The stability of the steady state is determined by the signs of the real parts of the eigenvalues *λ*_*i*_ of the associated eigenvectors **(Δx)**_*i*_ ≡ **L**_*i*_ of **H**, which are defined by the equation *λ*_*i*_ **L**_*i*_ = **L**_*i*_ **⋅ H** or

$$\begin{array}{c} L_{i}\cdot \left(\lambda_{i}I-H\right)=0. \end{array}$$ (4)

For a steady state to be stable, the real parts of all eigenvalues should be negative. For non-zero eigenvectors (**L**_*i*_ ≠ **0**), Equation 4 will only be true for singular (non-invertible) (*λ*_*i*_ **I**−**H**) having

$$\begin{array}{c} |\lambda I-H|=\rho (\lambda )=0. \end{array}$$ (5)

The roots of the characteristic polynomial *ρ*(*λ*) determine the eigenvalues and therefore the stability of the steady state:

$$\begin{array}{c} \rho \left(\lambda \right)=a_{0}\lambda^{n}+a_{1}\lambda^{n-1}+a_{2}\lambda^{n-2}+\cdots +a_{n-1}\lambda +a_{n}=0. \end{array}$$ (6)

While the first coefficient is equal to 1 by the above definition (Equation 5), we will retain the notation *a*_0_ below for clarity and generality. The coefficients *a*_*k*_ can be expressed as:

$$\begin{array}{cc} a_{k} & =\frac{1}{(n-k)!}{\left(\frac{\partial^{n-k}\rho }{\partial \lambda^{n-k}}\right)}_{\lambda =0}=\frac{1}{(n-k)!}{\left(\frac{\partial^{n-k}}{\partial \lambda^{n-k}}\left|\lambda I-H\right|\right)}_{\lambda =0}. \end{array}$$ (7)

It is clear that *a*_*n*_ = |−**H**| = (−1)^*n*^|**H**|. With a bit more effort [13], Equation 7 can be shown to entail:

$$\begin{array}{c} a_{q}={\left(-1\right)}^{q}b_{q}, \end{array}$$ (8)

with *b*_*q*_ representing the *q*×*q* principal minor of **H** (with *b*_0_ ≡ *a*_0_). Using the Leibniz rule, the principal minors of **H** can be written as:

$$\begin{array}{c} b_{q}=\sum_{i_{1}<\cdots <i_{q}}\sum_{\pi (j_{1},\ldots ,j_{q})}\epsilon_{j_{1}\ldots j_{q}}H_{i_{1}j_{1}}\cdots H_{i_{q}j_{q}}, \end{array}$$ (9)

with *π*(*j*_1_, …, *j*_*q*_) denoting the permutations of the ordered set {*i*_1_, …, *i*_*q*_} and *ϵ*_*j*_1_…*j*_*q*__ the Levi-Civita permutation symbol (equal to +1 for *j*_1_ = *i*_1_, …, *j*_*q*_ = *i*_*q*_ and otherwise equal to −1 for odd permutations or +1 for even permutations). While the above material can be found in standard references [6, 13], it is nevertheless presented here for completeness and for establishing important and slightly different notational conventions that will be used throughout this manuscript. For example, my unconventional expression of the network perturbation (Equation 3) as a perturbed species row vector multiplied on the right by the first-order transition matrix **H** was chosen to permit a convenient reading of the unipartite (species-only) *cycles* of the network from the (properly ordered) indices of the products of the *H*_*ij*_ (with *i* as usual denoting the row and *j* the column of **H**): for example, *H*_12_ *H*_23_ *H*_31_ corresponds to a 3-cycle from 1 → 2 then 2 → 3 then 3 → 1.

The principal minors have a simple topological interpretation as sums of (signed) products of all possible non-overlapping cyclic permutations. This is Sachs’ theorem, which was first derived in the early 1960’s (see [14] and references therein; see also Clarke’s simpler presentation of this elementary result [6]). Each principal minor, *b*_*q*_, corresponds to the sum of the product of all *unique* non-overlapping combinations of cycles, *c*_*l*_ (with cycle lengths *l* summing to *q*) in the network:

$$\begin{array}{ll} b_{1} & =\sum_{i}H_{ii}=c_{1},\\ b_{2} & =\frac{1}{2!}\sum_{i\neq j}H_{ii}H_{jj}-H_{ij}H_{ji}=\bar{c_{1}c_{1}}-c_{2},\\ b_{3} & =\frac{1}{3!}\sum_{i\neq j,i\neq k,j\neq k}H_{ii}H_{jj}H_{kk}\\ & -H_{ii}H_{jk}H_{kj}-H_{ik}H_{jj}H_{ki}-H_{ij}H_{ji}H_{kk}\\ & +H_{ij}H_{jk}H_{ki}+H_{ik}H_{ji}H_{ki}=\bar{c_{1}c_{1}c_{1}}-\bar{c_{1}c_{2}}+c_{3},\\ & \vdots \\ b_{q} & =\sum_{\begin{array}{c} 0\leq p_{1}\leq \ldots \leq p_{q}\\ \sum_{i}p_{i}=q \end{array}}{\left(-1\right)}^{s(p_{1},\ldots ,p_{q})}\bar{c_{p_{1}}c_{p_{2}}\ldots c_{p_{q}}}. \end{array}$$ (10)

The cycle term, *c*_*l*_, when appearing alone in the above expressions (i.e. without an overline), simply corresponds to the sum of all *l*-cycles in the network. The bar on top of a particular collection of cycle terms indicates the non-overlapping nature of the unipartite cycles in the product, i.e. each species can only appear at most once in a particular cycle product. For example, $\bar{c_{1}c_{2}}$ represents the sum of all unique, non-overlapping combinations of a 1-cycle and a 2-cycle in the network. In the final line, the *c*_0_ ≡ 1 are simply placeholders and *s*(*p*_1_, …, *p*_*q*_) is a function that returns the number of (non-zero) even length cycles (odd transpositions [6, 14]) present in the list {*p*_1_, …, *p*_*q*_}. In simpler terms, the principal minor *b*_*q*_ is the sum of all possible (cycle-based) partitions of *q*, with a negative sign accompanying each even cycle (odd transposition) in a given partition product. This topological definition of the principal minors is far more elegant and intuitive than the increasingly cumbersome index-based notation also displayed in the above for *b*_1_, *b*_2_ and *b*_3_.

While *a*_*q*_ > 0 for all *q* is necessary for stability, it is not *sufficient* [5]. The Routh-Hurwitz conditions [3–5], which are mathematically equivalent to the original criteria formulated by Hermite and the related criteria embodied in Lyapunov’s second method [9, 15–19], provide both necessary and sufficient conditions for steady-state stability. While these conditions have traditionally been obtained through the use of arcane mathematics, a remarkably simple proof has been found more recently requiring only basic algebra and continuity arguments [8]. The Routh-Hurwitz conditions for a stable steady state can be defined as:

$$\begin{array}{c} \Delta_{q}>0 \end{array}$$ (11)

for *q* = 1, …, *n*, with Δ_*q*_ denoting the Hurwitz determinant of the following matrix of the coefficients of the characteristic polynomial, *a*_*i*_, or the principal minors, *b*_*i*_ (see Equation 8):

$$\begin{array}{ccc} \Delta_{q} & = & \left|\begin{array}{cccccc} a_{1} & a_{0} & 0 & 0 & \cdots & 0\\ a_{3} & a_{2} & a_{1} & a_{0} & \cdots & 0\\ a_{5} & a_{4} & a_{3} & a_{2} & \cdots & 0\\ a_{7} & a_{6} & a_{5} & a_{4} & \cdots & 0\\ \vdots & \vdots & \vdots & \vdots & \ddots & \vdots \\ a_{2q-1} & a_{2q-2} & a_{2q-3} & a_{2q-4} & \cdots & a_{q} \end{array}\right|\\ & = & \left|\begin{array}{cccccc} -b_{1} & b_{0} & 0 & 0 & \cdots & 0\\ -b_{3} & b_{2} & -b_{1} & b_{0} & \cdots & 0\\ -b_{5} & b_{4} & -b_{3} & b_{2} & \cdots & 0\\ -b_{7} & b_{6} & -b_{5} & b_{4} & \cdots & 0\\ \vdots & \vdots & \vdots & \vdots & \ddots & \vdots \\ -b_{2q-1} & b_{2q-2} & -b_{2q-3} & b_{2q-4} & \cdots & {\left(-1\right)}^{q}b_{q} \end{array}\right|. \end{array}$$ (12)

The first few Hurwitz determinants in terms of the principal minors *b*_*q*_ are:

$$\begin{array}{ccc} \Delta_{1} & = & -b_{1} \end{array}$$ (13)

$$\begin{array}{ccc} \Delta_{2} & = & -b_{1}b_{2}+b_{0}b_{3} \end{array}$$ (14)

$$\begin{array}{ccc} \Delta_{3} & = & b_{1}b_{2}b_{3}-b_{0}b_{3}b_{3}+b_{0}b_{1}b_{5}-b_{1}b_{1}b_{4} \end{array}$$ (15)

$$\begin{array}{ccc} \Delta_{4} & = & b_{1}b_{2}b_{3}b_{4}-b_{0}b_{3}^{2}b_{4}-b_{1}^{2}b_{4}^{2}-b_{1}b_{2}^{2}b_{5}+b_{0}b_{2}b_{3}b_{5}+2b_{0}b_{1}b_{4}b_{5}\\ & & -\;b_{0}^{2}b_{5}^{2}+b_{1}^{2}b_{2}b_{6}-b_{0}b_{1}b_{3}b_{6}-b_{0}b_{1}b_{2}b_{7}+b_{0}^{2}b_{3}b_{7}. \end{array}$$ (16)

While the Liénard-Chipart conditions [5, 9, 20] are indeed simpler for determining the stability of a network steady state, the full Hurwitz determinants are more informative as they allow counting of the exact number of unstable roots via the Routh array (discussed below). Upon use of the purely cycle-based expressions for the principal minors (Equation 10), we obtain for the first two determinants:

$$\begin{array}{ccc} \Delta_{1} & = & -c_{1} \end{array}$$ (17)

$$\begin{array}{ccc} \Delta_{2} & = & -c_{1}\cdot \bar{c_{1}c_{1}}+c_{0}\cdot \bar{c_{1}c_{1}c_{1}}\\ & & +\;c_{1}\cdot c_{2}-c_{0}\cdot \bar{c_{1}c_{2}}\\ & & +\;c_{0}\cdot c_{3}. \end{array}$$ (18)

For a network with *n* ≤ 3 species, the terms that potentially contribute to the third determinant (corresponding to the first two terms of Equation 15) are:

$$\begin{array}{ccc} \Delta_{3} & = & c_{1}\cdot \bar{c_{1}c_{1}}\cdot \bar{c_{1}c_{1}c_{1}}-c_{0}\cdot \bar{c_{1}c_{1}c_{1}}\cdot \bar{c_{1}c_{1}c_{1}}\\ & & -\;c_{1}\cdot \bar{c_{1}c_{1}}\cdot \bar{c_{1}c_{2}}-c_{1}\cdot \bar{c_{1}c_{1}c_{1}}\cdot c_{2}+2c_{0}\cdot \bar{c_{1}c_{1}c_{1}}\cdot \bar{c_{1}c_{2}}\\ & & +\;c_{1}\cdot c_{2}\cdot \bar{c_{1}c_{2}}-c_{0}\cdot \bar{c_{1}c_{2}}\cdot \bar{c_{1}c_{2}}\\ & & +\;c_{1}\cdot \bar{c_{1}c_{1}}\cdot c_{3}-2c_{0}\cdot \bar{c_{1}c_{1}c_{1}}\cdot c_{3}\\ & & -\;c_{1}\cdot c_{2}\cdot c_{3}+2c_{0}\cdot \bar{c_{1}c_{2}}\cdot c_{3}\\ & & -\;c_{0}\cdot c_{3}\cdot c_{3}. \end{array}$$ (19)

In these expressions, *c*_0_ = *b*_0_ = 1 (*c*_0_, as used here, should not to be confused with the less meaningful “placeholder” *c*_0_ used to compute the cycle partitions in Equation 10). The raised dot indicates normal multiplication. Terms containing the same suite of cycle lengths are shown in the same line to indicate where cancellations can occur (e.g. in the top line of Equation 18 it is clear that the second term corresponding to the *positive* product of three non-overlapping 1-cycles, $\bar{c_{1}c_{1}c_{1}}$, will be canceled by the first term). Finding an appropriate topological notation that allows removal of all such potential cancellations, thereby reducing these expressions even further, remains an open problem [6, 7] (see as well the Discussion section below). Orlando’s formulas [5, 21] allow expression of the penultimate and ultimate Hurwitz determinants for a network with species dimensionality *n* as:

$$\begin{array}{ccc} \Delta_{n-1} & = & {\left(-1\right)}^{\frac{n(n-1)}{2}}a_{0}^{n-1}\prod_{1\leq i<k\leq n}\left(\lambda_{i}+\lambda_{k}\right) \end{array}$$ (20)

$$\begin{array}{ccc} \Delta_{n} & = & {\left(-1\right)}^{\frac{n(n-1)}{2}}a_{0}^{n}\lambda_{1}\ldots \lambda_{n}\prod_{1\leq i<k\leq n}\left(\lambda_{i}+\lambda_{k}\right). \end{array}$$ (21)

These formulas importantly indicate that Δ_*n*−1_ = Δ_*n*_ = 0 upon appearance of a pair of purely complex roots, representing a necessary condition for a Poincaré-Andronov-Hopf bifurcation [12] (referred to throughout this manuscript as a Hopf bifurcation). More generally, the number of unstable roots, *k*, with positive real part is equal to the number of sign changes in the first column of the Routh array, which appears below in the ordered arguments of the function *V*() [5]:

$$\begin{array}{c} k=V\left(a_{0},\Delta_{1},\frac{\Delta_{2}}{\Delta_{1}},\frac{\Delta_{3}}{\Delta_{2}},\ldots ,\frac{\Delta_{n}}{\Delta_{n-1}}\right). \end{array}$$ (22)

Here, *V*() merely returns the number of sign changes in the list contained within the parentheses when read from left to right. In the below, the arguments of *V*() will be expressed using only ‘+’ or ‘−’ signs. For example, *V*(+, −, +) has two sign changes and therefore two unstable eigenvalues. The above criterion clearly fails, or is ambiguous, if any of the Hurwitz determinants equals 0. For these cases, the following generalization must be taken [5]. Consider a consecutive string of *p* Hurwitz determinants that are all zero. If this string terminates at Δ_*n*_, then one can truncate the Routh array, applying the above criterion for the determinants Δ_1_, …, Δ_*n*−*p*_. If, however, Δ_*n*_ ≠ 0 (and therefore Δ_*n*−1_ ≠ 0, see Equations 20 and 21), with the string extending from Δ_*s*+1_, …, Δ_*s*+*p*_ (Δ_*s*_ ≠ 0 and Δ_*s*+*p*+1_ ≠ 0), then

$$\begin{array}{ccc} k & = & V\left(a_{0},\Delta_{1},\frac{\Delta_{2}}{\Delta_{1}},\ldots ,\frac{\Delta_{s}}{\Delta_{s-1}}\right)\\ & & +\;\frac{p+1}{2}+\frac{1}{2}\left(1-{\left(-1\right)}^{(p+1)/2}\mathrm{sign}\left(\frac{\Delta_{s}}{\Delta_{s-1}}\frac{\Delta_{s+p+2}}{\Delta_{s+p+1}}\right)\right)\\ & & +\;V\left(\frac{\Delta_{s+p+2}}{\Delta_{s+p+1}},\ldots ,\frac{\Delta_{n}}{\Delta_{n-1}}\right). \end{array}$$ (23)

The second line of the above is equal to (*p*+1)/2 if (−1)^(*p*+1)/2^ times the “sign” term yields +1, or (*p*+3)/2 if this product yields −1 (*p* will always be odd). For *s* = 0, the above formula should be modified to:

$$\begin{array}{ccc} k & = & \frac{p+1}{2}+\frac{1}{2}\left(1-{\left(-1\right)}^{(p+1)/2}\mathrm{sign}\left(a_{0}\frac{\Delta_{p+2}}{\Delta_{p+1}}\right)\right)\\ & & +\;V\left(\frac{\Delta_{p+2}}{\Delta_{p+1}},\ldots ,\frac{\Delta_{n}}{\Delta_{n-1}}\right). \end{array}$$ (24)

### 2 Expression of the Routh-Hurwitz Conditions Using the Influence Topology

Chemistry presents the interesting notion of a *reaction*, for which I give the following mathematical generalization (see also [1]). The *f*_*j*_(*x*_1_, …, *x*_*n*_) in Equation 1 can be expressed as the sum over *m* real reaction functions *v*_*k*_ multiplied by species-specific stoichiometric scalars $s_{j}^{k}$:

$$\begin{array}{c} f_{j}\left(x_{1},\ldots ,x_{n}\right)=\sum_{k=1}^{m}v_{k}s_{j}^{k}, \end{array}$$ (25)

with

$$\begin{array}{c} H_{ij}={\left(\frac{\partial f_{j}}{\partial x_{i}}\right)}_{s}=\sum_{k=1}^{m}\frac{\partial v_{k}}{\partial x_{i}}s_{j}^{k}. \end{array}$$ (26)

**Fig 1:**
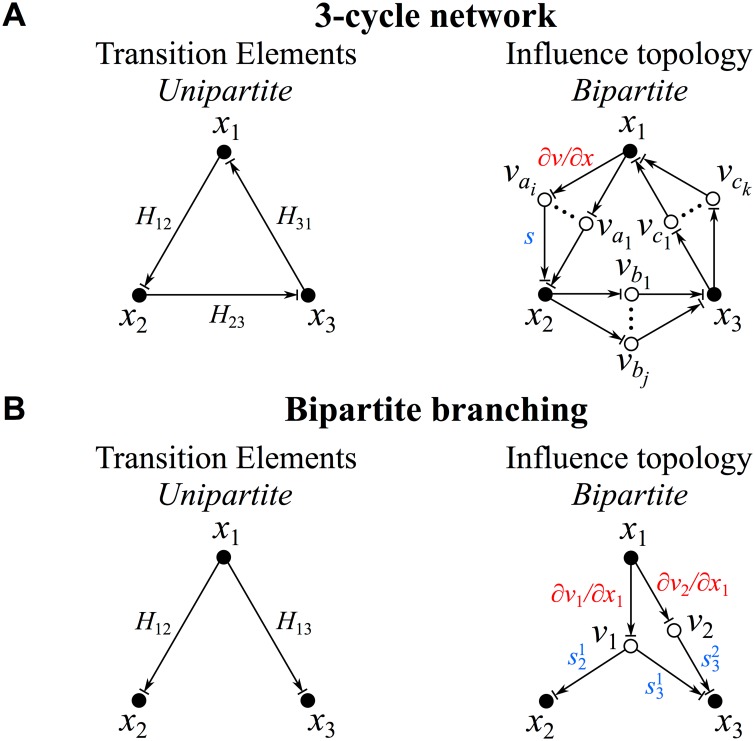
Unipartite versus bipartite representations. (A) Unipartite graph of a network comprised of a single 3-cycle; edges are labeled with the corresponding transition matrix elements of unspecified sign (left). The bipartite representation (influence topology) of this network is also displayed (right); here, species-to-reaction node edges correspond to the Jacobian elements (∂*v*/∂*x*) and reaction-to-species node edges correspond to the stoichiometries (*s*). (B) Unipartite versus bipartite depiction of a branching influence of one species on two immediately downstream species. See §2 for further details.

The transition elements therefore represent sums over products of reaction derivatives and stoichiometric scalars. While completely general, the above expression is of course not *unique* as it depends on the particular choice of definition of the network reactions (e.g. one could take the trivial, and not particularly helpful, choice of the reactions as corresponding directly to the *f*_*j*_, i.e. *f*_*j*_ = *v*_*j*_ for each *j*). The *v*_*k*_ represent completely arbitrary real functions of a subset of the *x*_*i*_. For the reaction derivatives ∂*v*_*k*_/∂*x*_*i*_ in the above definition of *H*_*ij*_, explicit reference to the particular steady state *s* has been dropped for notational convenience (both here and in all subsequent expressions and figures). The intrinsic *bipartite* nature of the reaction network topology has its fundamental basis in the separability of the *H*_*ij*_ transition elements into distinct *input* Jacobian terms (*i*) and *output* stoichiometry terms (*j*) for each reaction *k*, which serve as the basis of the network’s influence topology [1, 2] (see Fig. 1A and its further discussion below). It is clear from Equation 26 that the transition matrix can be expressed as the product of a Jacobian matrix and a stoichiometry matrix, yielding the following alternative form for Equation 3 of:

$$\begin{array}{ccc} \frac{d}{dt}\left(\Delta x_{1},\ldots ,\Delta x_{n}\right) & ≃ & \left(\Delta x_{1},\ldots ,\Delta x_{n}\right)\left(\begin{array}{ccc} \sum_{k}\frac{\partial v_{k}}{\partial x_{1}}s_{1}^{k} & \cdots & \sum_{k}\frac{\partial v_{k}}{\partial x_{1}}s_{n}^{k}\\ \vdots & \ddots & \vdots \\ \sum_{k}\frac{\partial v_{k}}{\partial x_{n}}s_{1}^{k} & \cdots & \sum_{k}\frac{\partial v_{k}}{\partial x_{n}}s_{n}^{k} \end{array}\right)\\ \frac{d}{dt}\left(\Delta x_{1},\ldots ,\Delta x_{n}\right) & ≃ & \left(\Delta x_{1},\ldots ,\Delta x_{n}\right)\left(\begin{array}{ccc} \frac{\partial v_{1}}{\partial x_{1}} & \cdots & \frac{\partial v_{m}}{\partial x_{1}}\\ \vdots & \ddots & \vdots \\ \frac{\partial v_{1}}{\partial x_{n}} & \cdots & \frac{\partial v_{m}}{\partial x_{n}} \end{array}\right)\left(\begin{array}{ccc} s_{1}^{1} & \cdots & s_{n}^{1}\\ \vdots & \ddots & \vdots \\ s_{1}^{m} & \cdots & s_{n}^{m} \end{array}\right). \end{array}$$ (27)

Banaji & Craciun [1, 2] consider the further generalization of the *f*_*j*_ as completely arbitrary functions of the reactions *v*_*k*_ (not simply a sum) through use of the chain rule, now with

$$\begin{array}{c} s_{j}^{k}\equiv \frac{\partial f_{j}}{\partial v_{k}} \end{array}$$ (28)

in Equation 26. In this case, the stoichiometry coefficients are now no longer global constants, but must be determined (along with the Jacobian reaction terms) at each particular steady state *s*. While mathematically interesting, the practical value of such further generalization remains unclear and is not further explored in the current manuscript where I will assume throughout the above definition of each *f*_*j*_ as a sum of reactions (Equation 25). It should nevertheless be noted though that the hierarchical algorithm for stability analysis developed in this manuscript carries over in a straightforward manner to this more general definition.

The principal minors of **H** (Equation 9) can now be rewritten as:

$$\begin{array}{ccc} b_{q} & = & \sum_{i_{1}<\cdots <i_{q}}\sum_{\pi (j_{1},\ldots ,j_{q})}\epsilon_{j_{1}\ldots j_{q}}H_{i_{1}j_{1}}\cdots H_{i_{q}j_{q}}\\ & = & \sum_{i_{1}<\cdots <i_{q}}\sum_{\pi (j_{1},\ldots ,j_{q})}\epsilon_{j_{1}\ldots j_{q}}\left(\sum_{k_{1}}\frac{\partial v_{k_{1}}}{\partial x_{i_{1}}}s_{j_{1}}^{k_{1}}\right)\cdots \left(\sum_{k_{q}}\frac{\partial v_{k_{q}}}{\partial x_{i_{q}}}s_{j_{q}}^{k_{q}}\right)\\ & = & \sum_{i_{1}<\cdots <i_{q}}\sum_{k_{1},\ldots ,k_{q}}\frac{\partial v_{k_{1}}}{\partial x_{i_{1}}}\cdots \frac{\partial v_{k_{q}}}{\partial x_{i_{q}}}\sum_{\pi (j_{1},\ldots ,j_{q})}\epsilon_{j_{1}\ldots j_{q}}s_{j_{1}}^{k_{1}}\cdots s_{j_{q}}^{k_{q}}, \end{array}$$ (29)

or, equivalently:

$$\begin{array}{ccc} b_{q} & = & \sum_{j_{1}<\cdots <j_{q}}\sum_{\pi (i_{1},\ldots ,i_{q})}\epsilon_{i_{1}\ldots i_{q}}H_{i_{1}j_{1}}\cdots H_{i_{q}j_{q}}\\ & = & \sum_{j_{1}<\cdots <j_{q}}\sum_{k_{1},\ldots ,k_{q}}s_{j_{1}}^{k_{1}}\cdots s_{j_{q}}^{k_{q}}\sum_{\pi (i_{1},\ldots ,i_{q})}\epsilon_{i_{1}\ldots i_{q}}\frac{\partial v_{k_{1}}}{\partial x_{i_{1}}}\cdots \frac{\partial v_{k_{q}}}{\partial x_{i_{q}}}. \end{array}$$ (30)

In both cases, it is clear that *k*_1_, …, *k*_*q*_ should all be distinct, otherwise they will cancel with related terms under the permutation, implying that each reaction will only appear once in each “surviving” product term defining a particular principal minor. As each term corresponds to a product of cycles, this leads to the following important topological generalization of the “non-overlapping” cycle products in Equation 10 to *bipartite* graphs (see also [1]): To avoid cancellations among the terms that comprise a particular principal minor, each bipartite cycle product should contain each species no more than once *and* each reaction no more than once. This is equivalent to the topological specification of non-overlapping bipartite cycles in the full *bipartite* graph of the network corresponding to its influence topology (discussed in greater detail below).

In Equation 29, specific stoichiometric subnetworks (defined by a particular species subset *i*_1_, …, *i*_*q*_ and reaction subset *k*_1_, …, *k*_*q*_) will not contribute if their determinant is zero (indicating that the basis vectors of the subnetwork span a volume of dimension lower than *q*). Mass conservation in only a partial graph of the subnetwork is sufficient to generate a zero determinant for the stoichiometric terms [22, 23]; however, there are many other ways that a zero determinant of the stoichiometry (indicating a conserved quantity) can be obtained. For example, for a chemical network with governing equations for the species concentrations of ${\dot{x}}_{1}=v_{1}$ and ${\dot{x}}_{2}=2v_{1}$, a zero determinant is obtained, with the difference in concentrations conserved (2*x*_1_−*x*_2_) not the total mass (e.g., *x*_1_+*x*_2_).

A zero determinant for the Jacobian matrix is also possible, but in practice rarer to obtain and very difficult to recognize based only on cursory inspection of the governing equations, as the values of the Jacobian reaction derivatives (for nonlinear reactions anyway) will generally differ at each steady state (unlike the constant stoichiometric matrix), with the exact locations of the steady states therefore also necessary to know.

We are now ready to more formally define the influence topology. Consider, in isolation, a cycle of length *l* that contributes to one of the cycle products indicated in Equation 10 and that connects in an ordered fashion the species *x*_*i*_1__ *x*_*i*_2__ *x*_*i*_3__…*x*_*i*_*l*__ *x*_*i*_1__:

$$\begin{array}{ccc} c_{l} & = & H_{i_{1}i_{2}}\cdots H_{i_{l}i_{1}}\\ & = & \left(\sum_{k_{1}}\frac{\partial v_{k_{1}}}{\partial x_{i_{1}}}s_{i_{2}}^{k_{1}}\right)\cdots \left(\sum_{k_{l}}\frac{\partial v_{k_{l}}}{\partial x_{i_{l}}}s_{i_{1}}^{k_{l}}\right)\\ & = & \sum_{k_{1},\ldots ,k_{l}}\left(\frac{\partial v_{k_{1}}}{\partial x_{i_{1}}}s_{i_{2}}^{k_{1}}\cdots \frac{\partial v_{k_{l}}}{\partial x_{i_{l}}}s_{i_{1}}^{k_{l}}\right). \end{array}$$ (31)

The last version constitutes a sum over all unique bipartite cycles over the ordered species. This is made explicit for a network comprised of a single 3-cycle in Fig. 1A, where the positive/negative (non-zero) effect of one species on another is illustrated by the superposition of an arrow (positive) and a blunt arrow (negative), the graphical equivalent of ‘±’. The 3-cycle defined by the unipartite product *H*_12_ *H*_23_ *H*_31_ in Fig. 1A is equivalent to the sum over all unique bipartite cycles (e.g. the bipartite cycles *x*_1_ *v*_*a*_1__ *x*_2_ *v*_*b*_1__ *x*_3_ *v*_*c*_1__ *x*_1_ and *x*_1_ *v*_*a*_2__ *x*_2_ *v*_*b*_1__ *x*_3_ *v*_*c*_1__ *x*_1_ provide distinct contributions due to their paths through the different reactions *v*_*a*_1__ and *v*_*a*_2__). That the bipartite graph conveys more complete information about the network than the corresponding unipartite graph is illustrated in Fig. 1B, in which the direct influence of one species on two other downstream species is depicted. For the unipartite graph, the branches to each individual species appear independent. For the bipartite version, a single reaction can affect both species. It is useful to restrict the notion of a *single* influence topology to one with fixed positive or negative signs at each edge, not the superposed positive/negative edges used in Fig. 1 (which implies a collection of influence topologies). As reaction-like terms provide a useful level of description of network dynamics in many different fields (see the below analysis of multiple classical networks), the signed directed bipartite graph corresponding to the influence topology provides the most complete representation of how a network of reactions communicates the influence of one species on its immediately downstream species in the important vicinity of a steady state solution.

### 3 Parameter Reduction of the Stability Phase Space

Reduction of the parameters required to describe a network’s stability not only represents a useful simplification, but also helps to reveal the truly important properties of a particular network that underlie its stability. In this section, three different possibilities for parameter reduction are presented: stoichiometric scaling, cycle compaction, and temporal scaling.

Stoichiometric scaling is best explained using the above expression of the transition matrix **H** as the product of the Jacobian matrix with the stoichiometric matrix (Equation 27). Without loss of generality, the rows of the stoichiometry matrix in Equation 27 can be scaled, with a corresponding inverse scaling of the Jacobian matrix columns, to obtain:

$$\begin{array}{ccc} \frac{d}{dt}\left(\Delta x_{1},\ldots ,\Delta x_{n}\right) & ≃ & \left(\Delta x_{1},\ldots ,\Delta x_{n}\right)\left(\begin{array}{ccc} \alpha_{1}\frac{\partial v_{1}}{\partial x_{1}} & \cdots & \alpha_{m}\frac{\partial v_{m}}{\partial x_{1}}\\ \vdots & \ddots & \vdots \\ \alpha_{1}\frac{\partial v_{1}}{\partial x_{n}} & \cdots & \alpha_{m}\frac{\partial v_{m}}{\partial x_{n}} \end{array}\right)\left(\begin{array}{ccc} \frac{s_{1}^{1}}{\alpha_{1}} & \cdots & \frac{s_{n}^{1}}{\alpha_{1}}\\ \vdots & \ddots & \vdots \\ \frac{s_{1}^{m}}{\alpha_{m}} & \cdots & \frac{s_{n}^{m}}{\alpha_{m}} \end{array}\right)\\ \frac{d}{dt}\left(\Delta x_{1},\ldots ,\Delta x_{n}\right) & ≃ & \left(\Delta x_{1},\ldots ,\Delta x_{n}\right)\left(\begin{array}{ccc} r_{1}^{1} & \cdots & r_{1}^{m}\\ \vdots & \ddots & \vdots \\ r_{n}^{1} & \cdots & r_{n}^{m} \end{array}\right)\left(\begin{array}{ccc} \sigma_{1}^{1} & \cdots & \sigma_{n}^{1}\\ \vdots & \ddots & \vdots \\ \sigma_{1}^{m} & \cdots & \sigma_{n}^{m} \end{array}\right), \end{array}$$ (32)

with

$$\begin{array}{ccc} \alpha_{k} & = & \left|s_{j_{k}}^{k}\right| \end{array}$$ (33)

$$\begin{array}{ccc} \sigma_{j}^{k} & = & \frac{1}{\alpha_{k}}\left|s_{j}^{k}\right| \end{array}$$ (34)

$$\begin{array}{ccc} r_{i}^{k} & = & \alpha_{k}\frac{\partial v_{k}}{\partial x_{i}}. \end{array}$$ (35)

**Fig 2:**
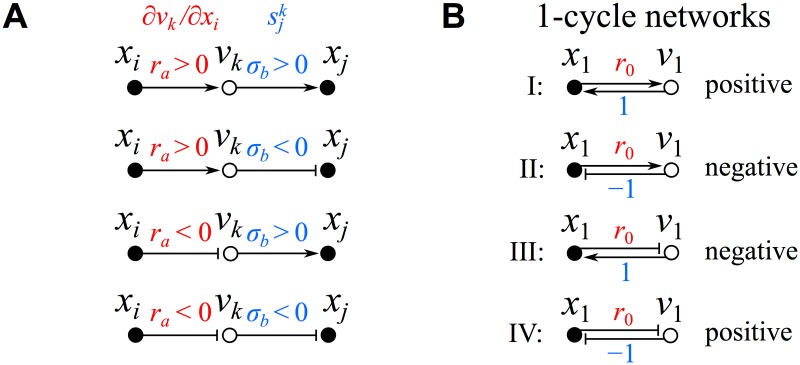
Species connectivity and 1-cycles. (A) All possible signed directed bipartite connections between two species. (B) All possible 1-cycle networks.

In the above, the *j*_*k*_ refer to a particular non-zero stoichiometry of reaction *k*. Scaling of the stoichiometry matrix merely amounts to a redefinition of the reactions such that at least one of the scaled stoichiometries of each reaction is ±1. For typical networks of interest, the ***σ*** matrix will be sparsely filled with elements that are either simply ±1 or the positive/negative (non-zero) real constants *σ*_1_, …, *σ*_*g*_. Similarly, the ***r*** matrix will be sparsely filled with elements equal to the positive/negative (non-zero) real numbers *r*_1_, …, *r*_*f*_ (*r*_0_ may also appear; see the discussion below). If the reaction has a strict monotonicity over the entire phase space, then we can assign this edge either an arrow (positive-definite monotonicity) or a blunt arrow (negative-definite) corresponding to a single fixed influence topology. If the monotonicity is not strict, this uncertainty in sign will be conveyed through the superposition of an arrow and a blunt arrow as discussed already above (see Fig. 1). The complete set of possible connections between two species using the above parameters is given in graphical terms in Fig. 2A with the related set of all possible 1-cycles given in Fig. 2B (discussed in greater detail in §4).

**Fig 3:**
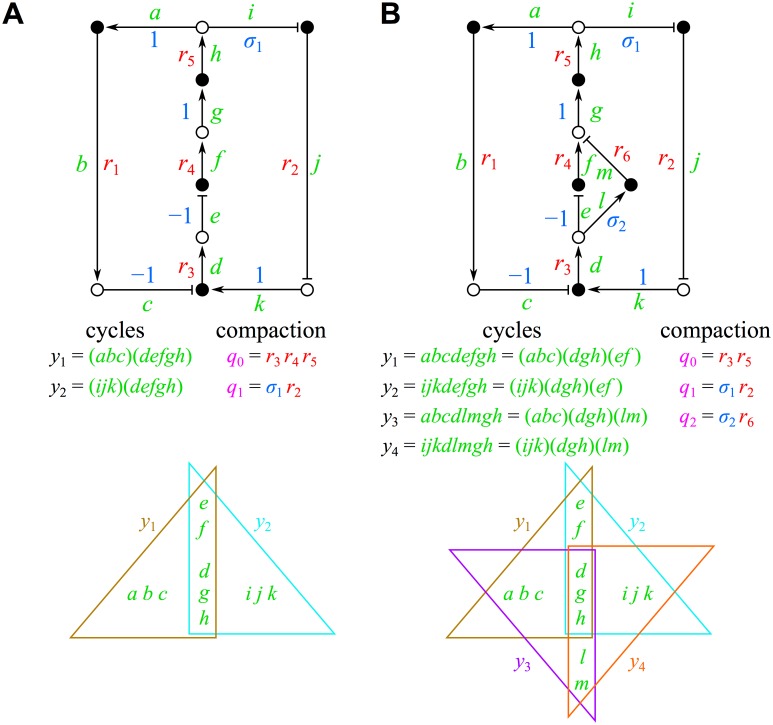
Cycle compaction. (A) For a network composed of two overlapping cycles, the possible cycle compaction terms (*q*_0_ and *q*_1_) are listed. (B) Upon slight modification of A, a network composed of four unique overlapping cycles is obtained, with now three possible compaction terms (*q*_0_, *q*_1_, and *q*_2_). The collection of edges that contribute to the non-overlapping and overlapping parts of the influence topology cycles for each network are shown as Venn diagrams at the bottom of each panel. See §3 for further details.

Individual *r*_*i*_ and *σ*_*j*_ terms appear in the Routh-Hurwitz conditions only through their contribution to complete cycles in the graph. Each cycle is simply the product of individual Jacobian and stoichiometric edges (see Equation 31 and Fig. 3). Additional parameter reduction can often be achieved through *cycle compaction*, which consists of the expression of a particular product of multiple *r*_*i*_ and/or *σ*_*j*_ terms as a single cycle compaction term *q*_*k*_. Cycle compaction can be easily understood from either an algebraic or a topological perspective (see Fig. 3). From an algebraic perspective, write down all cycles in the graph in terms of their products of Jacobian and stoichiometric edges. Certain combinations of these factors may always appear together, allowing their replacement by a single *q*_*k*_ variable (*q*_1_, *q*_2_, …). From a topological perspective, draw all directed cycles in the network: Each edge will be present in one or more cycles. For a particular overlap of a certain set of cycles (and no other cycles), the multiple edges that define this overlap can be compacted into a single parameter *q*_*k*_. Consider the following binary “bar code” for defining a particular region of overlap among several cycles, with the first entry of 0 or 1 corresponding to the presence/absence of an overlap with cycle 1, the second entry of 0 or 1 to cycle 2, etc. There are clearly 2^*c*^−1 possible types of overlap among *c* cycles, providing an important upper limit to the dimensionality of the set of parameters that govern the network’s steady-state stability. This list of overlaps includes the non-overlapping portions of each cycle; the −1 in the above removes the trivial bar code of all zeroes corresponding to the empty set. As shown in Fig. 3B, compacted regions of overlap (or non-overlap) in the influence topology need not be contiguous. At a deeper level, cycle compaction terms provide a fundamental basis for expression of the cycles of the network, implying that these are the underlying variables upon which the Hurwitz determinants ultimately depend (see the Discussion section).

A final degree of freedom can always be removed by temporal scaling, *τ* = *βt*, with *β* = |*r*_0_| (scaling to a particular Jacobian element *r*_0_) or *β* = |*q*_0_|^1/*z*^ (scaling to a particular cycle compaction term *q*_0_ containing *z* ≥ 1 Jacobian elements):

$$\begin{array}{c} \;\frac{d}{d\tau }\left(\Delta x_{1},\ldots ,\Delta x_{n}\right)≃\left(\Delta x_{1},\ldots ,\Delta x_{n}\right)\left(\begin{array}{ccc} \rho_{1}^{1} & \cdots & \rho_{1}^{m}\\ \vdots & \ddots & \vdots \\ \rho_{n}^{1} & \cdots & \rho_{n}^{m} \end{array}\right)\left(\begin{array}{ccc} \sigma_{1}^{1} & \cdots & \sigma_{n}^{1}\\ \vdots & \ddots & \vdots \\ \sigma_{1}^{m} & \cdots & \sigma_{n}^{m} \end{array}\right), \end{array}$$ (36)

with $\rho_{i}^{k}=r_{i}^{k}/\beta$. The subscript 0 on *r*_0_ or *q*_0_ will be used in all of the below graphs of the influence topology to indicate which term is used for temporal scaling (itself contributing ±1); this term is retained in the graph to stress the arbitrary nature of this choice. The above ***ρ*** matrix will therefore be sparsely filled with one ±1 and *ρ*_1_, …, *ρ*_*f*−1_ (scaling to *r*_0_) or *ρ*_1_, …, *ρ*_*f*_ (scaling to *q*_0_). Again, each *ρ*_*i*_ should be considered strictly positive (arrow) or strictly negative (blunt arrow) for the definition of a single influence topology.

**Fig 4:**
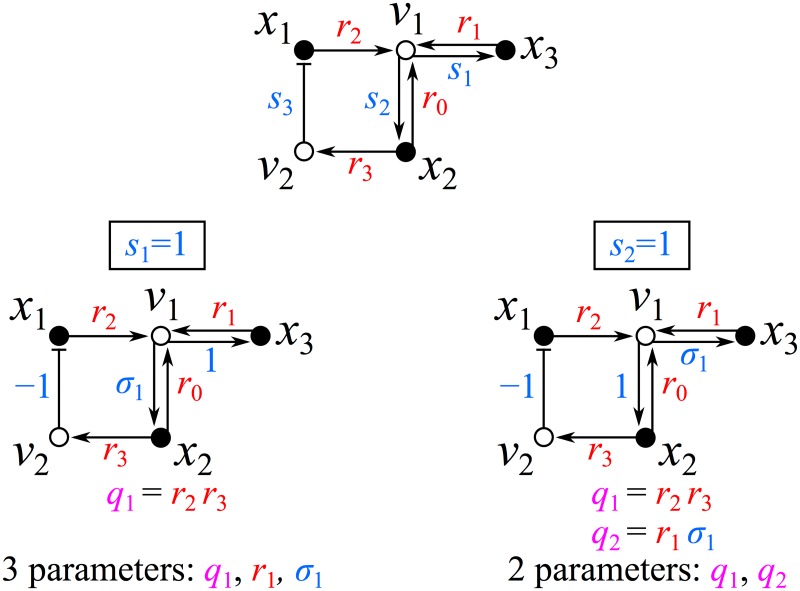
Non-redundancy of stoichiometric reduction and cycle compaction. The displayed network (top) provides a concrete example of the non-redundancy of stoichiometric reduction and cycle compaction. At the top, all Jacobian parameters (*r*_0_ = 1, *r*_1_, *r*_2_, *r*_3_) and stoichiometric parameters (*s*_1_, *s*_2_, *s*_3_) of the network are labeled. Stoichiometric reduction of reaction *v*_1_ allows the setting of either *s*_1_ (bottom left) or *s*_2_ (bottom right) to unity. The choice of *s*_1_ = 1 followed by cycle compaction leads to three final parameters (3D stability phase space), whereas the better choice of *s*_2_ = 1 leads to only two final parameters (2D stability phase space).

The first-order stability of a network of *n* species and *m* reactions at a particular steady state is determined by the *d* = *S*+*J* parameters that respectively define its total number of stoichiometric edges, *S*, and Jacobian edges, *J*. As already discussed above, stoichiometric scaling allows one stoichiometric factor for each reaction to be set to ±1, with the others labeled as *σ*_1_, …, *σ*_*S*−*m*_. Cycle compaction, which should be performed in concert with stoichiometric scaling, can provide an additional reduction of *c* dimensions. While stoichiometric reduction and cycle compaction are often redundant, the potential independence of stoichiometric reduction and cycle compaction for certain networks is demonstrated in Fig. 4. Finally, temporal scaling generically allows removal of an additional degree of freedom, giving a final dimensionality of *d* = (*S*−*m*)+*J*−*c*−1. It is worth emphasizing that all of these parameter reductions were achieved purely through examination of the network’s influence topology. Based on the reduced parameters of the influence topology (with fixed sign), the zones over which each of the Hurwitz determinants are negative (indicating instability) can be plotted over the entire parameter domain. This permits lower dimensional visualization of the complete stability phase space entailed by a particular influence topology. The stability phase space importantly delimits the set of possible dynamical solutions of networks sharing a particular influence topology, providing the exact number of unstable roots (eigenvalues) via the Routh array in each zone (and zone overlap) pertaining to the individual Hurwitz determinants. The utility of the stability phase space for the quantitative assessment of the stability of several classical networks is demonstrated below (see §5).

Other potentially useful forms of parameter reduction are possible through symmetries in the influence topology graph. Exchanges of certain subsets of the *r*_*i*_ and *σ*_*j*_ can be topologically shown to generate an identical set of non-overlapping cycle products that define the principal minors (*topological symmetry*), allowing redefinition of multiple parameters into a single parameter, Ψ, which is symmetric with respect to the *r*_*i*_ and/or *σ*_*j*_ parameters that define it. As well, the complex algebraic structure of the Hurwitz determinant inequalities can allow for the algebraic gathering of multiple parameters into one parameter (*Hurwitz reduction*), often allowing expression of the stability of an entire network in terms of an inequality involving a single parameter, Γ. It is possible that these Hurwitz reductions also have a topological explanation, but not one as simple as all of the others described above. Examples of networks containing topological symmetries or Hurwitz reductions will be encountered below.

The following generic graphical features, which I refer to as orphan/childless species and orphan/childless reactions, appear upon consideration of arbitrary directed bipartite graphs (see also [1]). These features will appear again in the more general discussion of network analysis presented below in §6.

“Orphan species” are those that do not have any reactions as parents, i.e. they do not lie downstream of any reaction node in the influence topology. They often appear explicitly in chemical reaction networks as species buffered by an infinite bath (*clamped* species). Orphan species have the same mathematical status as a change in the coefficients governing the description of the reactions and can therefore be safely removed from the graph of the influence topology.

“Childless species” are those that are not the parent of any reaction, i.e. they do not lie upstream of any reaction node in the influence topology. Such species play only a “bookkeeping” role (e.g. to account for mass conservation), with no effect on the network’s dynamics and can therefore be neglected in the graph of the influence topology: The species *j* that comprise the relevant set of ODE’s in Equation 1 should be restricted to only those that affect other species in the network.

“Orphan reactions” are reactions that have no species as their parents, i.e. they do not lie downstream of any species node in the influence topology and therefore have no functional dependence on any of the species. Orphan reactions are mathematically equivalent to the addition of a (possibly different) constant term to one or more of the governing the network (see Equations 1 and 25). While they have no effect on the influence topology (as they are removed upon taking the first derivatives of the governing equations *f*_*j*_(*x*_1_, …, *x*_*n*_)), orphan reactions can nevertheless shift the location of the steady states within the stability phase space defined by the influence topology, potentially generating bifurcations in the network’s dynamics (e.g. Hopf bifurcation). For this reason, they will be retained in the graphs of specific networks considered below as a single *V* ^0^ node with one or more dashed lines (having possibly different stoichiometries) connected to the relevant species.

“Childless reactions” are reactions that have no species as children in the network under consideration. While they will never appear when defining the influence topology corresponding to a given set of ODEs, they nevertheless appear in the list of all possible directed bipartite graphs. Such reactions, while possibly controlled by a subset of the species of the network under consideration (with which they would share a Jacobian edge), can have no possible effect on the dynamics of these species and can therefore be removed from the influence topology graph. They can nevertheless affect other purely downstream species not under direct consideration. Such upstream/downstream partitioning of a network will take on a broader meaning in the discussion of general network analysis given below (see §6).

### 4 *n*-cycle networks

The simplest networks to analyze are those comprised of a single cycle of length *n*. For odd *n*, I show below that the Routh array allows determination of the exact number of unstable roots.

The simplest possible networks are constructed from a single 1-cycle. The four possible 1-cycle networks are:

$$\begin{array}{ccc} I:\;{\dot{x}}_{1} & = & \pm V^{0}+V_{1}^{1} \end{array}$$ (37)

$$\begin{array}{ccc} \mathrm{II}:\;{\dot{x}}_{1} & = & \pm V^{0}-V_{1}^{1} \end{array}$$ (38)

$$\begin{array}{ccc} \mathrm{III}:\;{\dot{x}}_{1} & = & \pm V^{0}+V_{\bar{1}}^{1} \end{array}$$ (39)

$$\begin{array}{ccc} \mathrm{IV}:\;{\dot{x}}_{1} & = & \pm V^{0}-V_{\bar{1}}^{1}, \end{array}$$ (40)

with the different reaction functions considered as positive definite and the reduced topological representation for these networks given in Fig. 2B (stoichiometric scaling leads to a ±1 stoichiometric edge with temporal scaling by |*r*_0_| implies a ±1 Jacobian edge; alternatively, and more fundamentally, cycle compaction and temporal scaling allows assigning the single number *q*_0_ = ±1 to the network). In the above, I introduce the following useful shorthand notation $V_{i_{1}\ldots i_{h}}^{k}\equiv v_{k}(x_{i_{1}},\ldots ,x_{i_{h}})$. The subscripts in $V_{i_{1}\ldots i_{h}}^{k}$ indicate a monotically increasing (normal subscript) or decreasing (overlined subscript) dependence on the *h* different species that control the reaction; an underlined subscript will be used to indicate an uncertain sign of the monotonicity. Due to the *n* = 1 dimensionality, only the first Routh-Hurwitz condition, Δ_1_ = −*b*_1_ = −*c*_1_, is necessary to consider for the above 1-cycle networks, giving for networks I-IV, respectively, *b*_1_ = 1, −1, −1, 1 and Δ_1_ = −1, 1, 1, −1. Networks I and IV are therefore unstable and networks II and III are stable. Note that only the signs of the reaction stoichiometry and its monotonicity with respect to *x*_1_ are necessary to specify to ascertain the network’s stability. Addition of the static terms ±*V* ^0^ can shift the steady-state solution but cannot otherwise affect the dynamics. For a reaction function $V_{\underline{1}}^{1}=v_{1}(x_{1})$ that does not have a strict monotonicity (either increasing or decreasing with respect to *x*_1_), the *x*_1_ phase space can be partitioned into regions over which either $V_{1}^{1}$ or $V_{\bar{1}}^{1}$ holds, corresponding to a single influence topology in each region (this can of course be generalized to higher dimensional phase spaces as well). A simple explicit example of networks I–IV is given below:

$$\begin{array}{ccc} I:\;{\dot{x}}_{1} & = & -1+x_{1} \end{array}$$ (41)

$$\begin{array}{ccc} \mathrm{II}:\;{\dot{x}}_{1} & = & 1-x_{1} \end{array}$$ (42)

$$\begin{array}{ccc} \mathrm{III}:\;{\dot{x}}_{1} & = & -1+1/x_{1} \end{array}$$ (43)

$$\begin{array}{ccc} \mathrm{IV}:\;{\dot{x}}_{1} & = & 1-1/x_{1}. \end{array}$$ (44)

For all of these networks, additional constant terms have been added to position the single steady state solution at the positive value of $x_{1}^{s}=1$. Networks I and II correspond to the familiar examples of exponential growth and decay, respectively. Networks III and IV are perhaps more exotic, but, from the perspective of the influence topology, are equally fundamental. For these examples, I have chosen the particularly simple reaction functions proportional to *x*_1_ and 1/*x*_1_, but any functions having the same stoichiometric sign and reaction monotonicity will have the same stability/instability (e.g. one could replace *x*_1_ with $e_{1}^{x}$ in network I or 1/*x*_1_ with 1/arctan *x*_1_ in network III).

**Fig 5:**
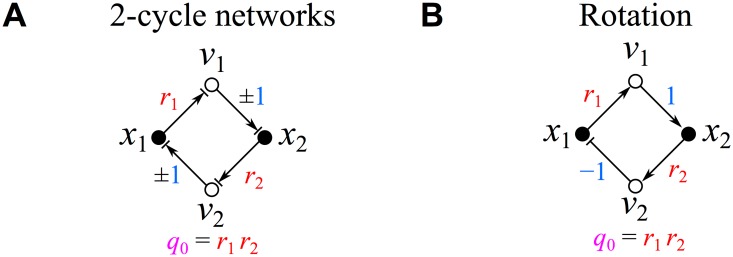
2-cycle networks. (A) All possible 2-cycle network influence topologies. (B) Rotation network influence topology.

All possible 2-cycle networks are schematically represented in the single graph shown in Fig. 5 (the degeneracy of these networks will be addressed further below). After cycle compaction (defining *q*_0_ = *r*_1_ *r*_2_) and temporal scaling ($\rho_{1}=r_{1}/\sqrt{\left|q_{0}\right|}$ and $\rho_{2}=r_{2}/\sqrt{\left|q_{0}\right|}$), it is clear that *b*_1_ = 0 and *b*_2_ = −*c*_2_ = ∓1. For all of these topologies, it is obvious that Δ_1_ = −*b*_1_ = 0 and Δ_2_ = −*b*_1_ *b*_2_+*b*_0_ *b*_3_ = 0 due to the absence of 1-cycles in the network. That all Routh-Hurwitz conditions are equal to 0 implies that no information can be obtained from first-order perturbations about the steady state; higher order perturbations must be assessed to establish the stability of a given steady state. An important example of a 2-cycle network is:

$$\begin{array}{c} {\dot{x}}_{1}=-k_{1}x_{2} \end{array}$$ (45)

$$\begin{array}{c} {\dot{x}}_{2}=k_{2}x_{1}, \end{array}$$ (46)

which, for *k*_1_ = *k*_2_, corresponds to constant rotational motion at a fixed radius determined by the initial values (boundary conditions). We can rewrite the rotation network in a more general way as:

$$\begin{array}{c} {\dot{x}}_{1}=-V_{2}^{1} \end{array}$$ (47)

$$\begin{array}{c} {\dot{x}}_{2}=V_{1}^{2}. \end{array}$$ (48)

In the above, I again employ the shorthand notation for the reaction functions explained above, with $V_{i}^{k}$ corresponding to reaction *k* with positive monotonic dependence on species *i*. The principal minors for this generalized network are *b*_1_ = 0 and *b*_2_ = −*c*_2_ = 1, which, as for the general case, leads to Δ_1_ = 0 and Δ_2_ = 0 and no information about steady state stability obtainable at first order. For the original rotation network (Equations 45 and 46), the linearity of the reactions implies that all higher order perturbations are trivially 0. The different solutions of this network depend on the initial conditions and foliate the *x*_1_-*x*_2_ phase space as circles of each possible radius centered on the origin. Inclusion of non-zero constant terms (*V* ^0^ terms) would merely shift the origin of these foliated circular trajectories.

According to Equation 10, a network comprised of a single *n*-cycle will yield *b*_*n*_ = *c*_*n*_ for odd *n* and *b*_*n*_ = −*c*_*n*_ for even *n* with all other principal minors equal to 0. For even *n*, examination of the non-zero terms in the columns of the Routh-Hurwitz matrix (Equation 12) shows that columns containing *b*_0_ = 1 and −*b*_*n*_ alternate with all-zero columns, implying that all Hurwitz determinants equal 0, with no further information possible at first-order (the generalization of the above result obtained for 2-cycles). This result can actually be generalized further: For an influence topology comprised of only even cycles, all Hurwitz determinants are zero, implying no information is obtainable at first order. For odd *n* ≥ 3 cycle networks, column swapping of the Hurwitz determinants (Equation 12) to place the single non-zero term in each column (either *b*_0_ = 1 or −*b*_*n*_) along the diagonal can be shown to lead to the following general result:

$$\begin{array}{ccc} \Delta_{1} & = & 0\\ & \vdots \\ \Delta_{n-2} & = & 0\\ \Delta_{n-1} & = & {\left(-1\right)}^{\frac{n+1}{2}}c_{n}^{\frac{n-1}{2}} \end{array}$$ (49)

$$\begin{array}{ccc} \Delta_{n} & = & -{\left(-1\right)}^{\frac{n+1}{2}}c_{n}^{\frac{n+1}{2}}. \end{array}$$ (50)

After cycle compaction and temporal scaling, *c*_*n*_ = ±1. For *n* = 3, Δ_2_ = ±1 and Δ_3_ = −1; for *n* = 5, Δ_4_ = −1 and Δ_5_ = ±1. For *n* = 7, this pattern repeats with Δ_6_ = ±1 and Δ_7_ = −1. From the Routh array (Equation 24), it can easily be shown that the number of unstable roots for an odd *n*-cycle network is:

$$\begin{array}{c} k=\frac{1}{2}\left(n+{\left(-1\right)}^{(n-1)/2}c_{n}\right). \end{array}$$ (51)

For *n* = 3, 7, 11, …, this implies *k* = (*n*−1)/2 for *c*_*n*_ = 1 and *k* = (*n*+1)/2 for *c*_*n*_ = −1. Oppositely, for *n* = 5, 9, 13, …, the above implies *k* = (*n*−1)/2 for *c*_*n*_ = −1 and *k* = (*n*+1)/2 for *c*_*n*_ = 1.

### 5 Analysis of classical networks

In the following, I provide detailed examinations of six classical networks from the diverse fields of control theory (Jenkin-Maxwell [24]), electronics (van der Pol [25]), ecology (Lotka-Volterra [26–28]), chemistry (Brusselator [29]), biochemistry (Sel’kov [30]), and synthetic biology (Repressilator [31–34]). Each network is generalized to its parameter reduced influence topology, with its full stability phase space examined for regions in which one or more Hurwitz determinants are negative. Particular attention is paid to those regions in which both Hurwitz determinants Δ_*n*−1_ and Δ_*n*_ simultaneously go negative, a necessary condition for the presence of a Hopf bifurcation (see §1). For most of the networks, an explicit expression of the steady state solution for the original governing equations allows display of the actually accessible regions of the stability phase space. These examples raise several important issues discussed in greater detail in the Discussion section.

#### Jenkin-Maxwell network

In Maxwell’s foundational paper on control theory from 1868 entitled “On Governors” [24], he considered several examples of physical devices that worked to *govern*—and, importantly, sustain against perturbation—the angular velocity of a core component. For one such physical device described by Jenkin, Maxwell derived the following second order differential equations:

$$\begin{array}{ccc} B\frac{d^{2}y}{dt^{2}} & = & F\left(\frac{dx}{dt}-V_{1}\right)-Y\frac{dy}{dt}-W \end{array}$$ (52)

$$\begin{array}{ccc} M\frac{d^{2}x}{dt^{2}} & = & P-R-F\left(\frac{dx}{dt}-V_{1}\right)-Gy. \end{array}$$ (53)

**Fig 6:**
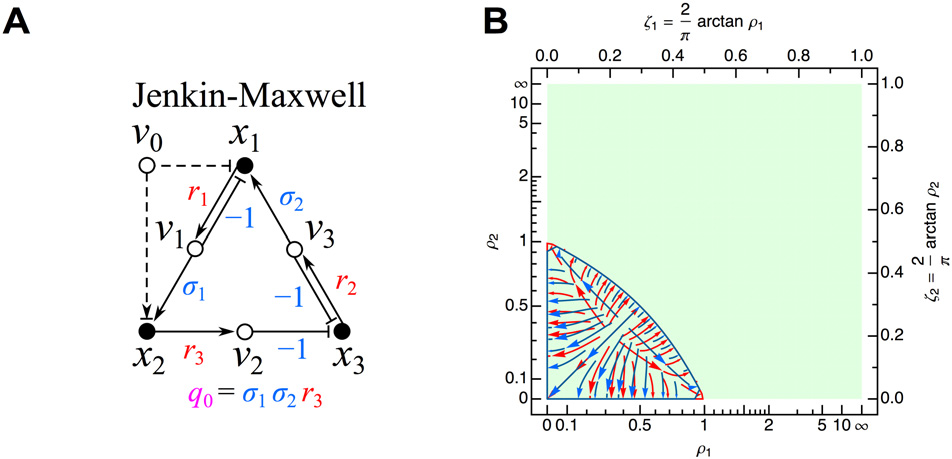
Jenkin-Maxwell network. (A) Influence topology. Cycle compaction allows definition of *q*_0_ = *σ*_1_ *σ*_2_ *r*_3_. Temporal scaling of all Jacobian edges to |*q*_0_| leaves only *ρ*_1_ = *r*_1_/|*q*_0_| and *ρ*_2_ = *r*_2_/|*q*_0_|. (B) Stability phase space. Axes correspond to the two parameters, *ρ*_1_ and *ρ*_2_, that remain after parameter reduction. For plotting *ρ*_1_ and *ρ*_2_, the variable transformation $\zeta_{i}=\frac{2}{\pi }\arctan\rho_{i}$ has been used to allow visualization of the entire range of the *ρ*_*i*_ from 0 to ∞ (this arctan transform also conveniently permits visualization of the range −∞ < *ρ*_*i*_ < 0, which would correspond to a different sign for this Jacobian element and therefore a different influence topology). Flows in the plot map the zones over which Δ_1_ (black), Δ_2_ (red), and Δ_3_ (blue) are negative. Only Δ_2_ (red) and Δ_3_ (blue) can go negative (in this case, simultaneously). The green background indicates that *ρ*_1_ and *ρ*_2_ can independently assume any positive definite values based on their definitions in terms of the parameters used to define the original Jenkin-Maxwell equations (Equations 54–56).

In the above, the nine parameters *B*, *F*, *V*_1_, *Y*, *W*, *M*, *P*, *R*, and *G* are all positive definite. Taking *x*_1_ = *dy*/*dt*, *x*_2_ = *y*, and *x*_3_ = *dx*/*dt*, these two second order equations reduce to the following three first-order equations:

$$\begin{array}{c} {\dot{x}}_{1}=-k_{0}\;\;\;-k_{1}x_{1}\;\;\;+\sigma_{2}k_{2}x_{3} \end{array}$$ (54)

$$\begin{array}{c} {\dot{x}}_{2}=\sigma_{1}k_{1}x_{1} \end{array}$$ (55)

$$\begin{array}{c} {\dot{x}}_{3}=\pm \sigma_{3}k_{0}\;\;\;-k_{2}x_{3}\;\;\;-k_{3}x_{2}, \end{array}$$ (56)

with *k*_0_ = (*FV*_1_+*W*)/*B*, *k*_1_ = *Y*/*B*, *σ*_2_ = *M*/*B*, ±*σ*_3_ *k*_0_ = (*FV*_1_+*P*−*R*)/*M*, *k*_2_ = *F*/*M*, and *k*_3_ = *G*/*M*. The generalized Jenkin-Maxwell network is:

$$\begin{array}{c} {\dot{x}}_{1}=-V^{0}\;\;\;-V_{1}^{1}\;\;\;+\sigma_{2}V_{3}^{2} \end{array}$$ (57)

$$\begin{array}{c} {\dot{x}}_{2}=\sigma_{1}V_{1}^{1} \end{array}$$ (58)

$$\begin{array}{c} {\dot{x}}_{3}=\pm \sigma_{3}V^{0}\;\;\;-V_{3}^{2}\;\;\;-V_{2}^{3}, \end{array}$$ (59)

corresponding to the influence topology shown in Fig. 6A, with principal minors:

$$\begin{array}{c} b_{1}=c_{1}\;\;\;=-\rho_{1}-\rho_{2} \end{array}$$ (60)

$$\begin{array}{c} b_{2}=\bar{c_{1}c_{1}}-c_{2}\;\;\;=\rho_{1}\rho_{2} \end{array}$$ (61)

$$\begin{array}{c} b_{3}=\bar{c_{1}c_{1}c_{1}}-\bar{c_{1}c_{2}}+c_{3}\;\;\;=-\rho_{1}\rho_{2}. \end{array}$$ (62)

In the above, *ρ*_1_ = *r*_1_/|*q*_0_| and *ρ*_2_ = *r*_2_/|*q*_0_| with *q*_0_ = *σ*_1_ *σ*_2_ *r*_3_. The principal minor *b*_1_ is given by the sum of the two 1-cycles: (*r*_1_/|*q*_0_|)(−1) = −*ρ*_1_ and (*r*_2_/|*q*_0_|)(−1) = −*ρ*_2_. For *b*_2_, only the first term (corresponding to two non-overlapping 1-cycles) contributes due to the absence of a 2-cycle in the network. For *b*_3_, only the 3-cycle contributes as there are only two non-overlapping 1-cycles (not three) and as there is no 2-cycle in the network. The 3-cycle is (*r*_1_/|*q*_0_|)*σ*_1_(*r*_3_/|*q*_0_|)(−1)(*r*_2_/|*q*_0_|)*σ*_2_ = −*ρ*_1_ *ρ*_2_. The Hurwitz determinants, determined from the above principal minors using Equations 13–15, are:

$$\begin{array}{ccc} \Delta_{1} & = & \rho_{1}+\rho_{2} \end{array}$$ (63)

$$\begin{array}{ccc} \Delta_{2} & = & \rho_{1}\rho_{2}\left(\rho_{1}+\rho_{2}-1\right) \end{array}$$ (64)

$$\begin{array}{ccc} \Delta_{3} & = & \rho_{1}^{2}\rho_{2}^{2}\left(\rho_{1}+\rho_{2}-1\right). \end{array}$$ (65)

One can also derive these determinants directly from the cycles in the graph using Equations 17–19, with the particular topology of the Jenkin-Maxwell network (two non-overlapping 1-cycles, no 2-cycles, one 3-cycle) leading to the following reduced form:

$$\begin{array}{ccc} \Delta_{1} & = & -c_{1} \end{array}$$ (66)

$$\begin{array}{ccc} \Delta_{2} & = & -c_{1}\cdot \bar{c_{1}c_{1}}+c_{0}\cdot c_{3} \end{array}$$ (67)

$$\begin{array}{ccc} \Delta_{3} & = & c_{1}\cdot \bar{c_{1}c_{1}}\cdot c_{3}-c_{0}\cdot c_{3}\cdot c_{3}. \end{array}$$ (68)

Upon plugging in for the cycles, the same expressions in Equations 63–65 obtain. The only negative term in Δ_2_ is *c*_0_⋅*c*_3_ = −*ρ*_1_ *ρ*_2_. In Δ_3_, the only negative term is $-c_{3}\cdot c_{3}=-\rho_{1}^{2}\rho_{2}^{2}$. These are the critical *multiplicative topologies* present in the influence topology. A multiplicative topology is simply a product of multiple subgraphs (overlapping or non-overlapping) of the network. The notion of multiplicative topology captures much better the true nature of the destabilizing structures in the graph than previous notions of *critical fragments*, which have in the past been typically based on the non-overlapping cycle products that contribute to the negativity of a particular principal minor, which constitutes only a sufficient but not necessary condition for instability.

The stability phase space is displayed in Fig. 6B. As *ρ*_1_ and *ρ*_2_ are both assumed positive, the condition for stability can be summarized as:

$$\begin{array}{c} \rho_{1}+\rho_{2}-1>0, \end{array}$$ (69)

or, for Ψ ≡ *ρ*_1_+*ρ*_2_ (with Ψ strictly positive), simply Ψ > 1. This additional reduction of the problem to a single parameter arises from the symmetric contributions of *r*_1_ and *r*_2_ to the principal minors (swapping of *r*_1_ and *r*_2_ in Fig. 6A would lead to the same criterion). For *ρ*_1_+*ρ*_2_−1 < 0, both Δ_2_ and Δ_3_ are negative, giving two unstable roots according to the number of sign changes in the Routh array *V*(+, +, −, +) (Equation 22).

For the specific Jenkin-Maxwell network defined by the parameters of Equations 52 and 53, *ρ*_1_ = *Y* ^2^/(*GB*) and *ρ*_2_ = *FY*/(*GM*), which, due to the simple linear dependence of the reactions on the species in Equations 54–56, are independent of the exact location of the single steady-state solution, which, at any rate, is located at $x_{1}^{s}=0$, $x_{2}^{s}=(P-R-W)/G$, and $x_{3}^{s}=V_{1}+W/F$. Using the above definitions of *ρ*_1_ and *ρ*_2_, the condition for steady-state stability becomes:

$$\begin{array}{c} \frac{Y^{2}}{GB}+\frac{FY}{GM}-1>0. \end{array}$$ (70)

Upon multiplication by the positive constant *G*/*B*, this is identical to the stability criterion obtained by Maxwell through explicit solution of the roots of the cubic characteristic polynomial. Such explicit algebraic solution is impossible for networks (and their associated characteristic polynomials) that have dimension *n* > 4; however, due to the remarkable properties of the Routh-Hurwitz conditions, in these situations one can still derive similarly strong topological/algebraic constraints through use of the influence topology. The green background in Fig. 6B indicates that *ρ*_1_ and *ρ*_2_, according to the definitions above, can assume any positive definite values. For *ρ*_1_+*ρ*_2_−1 > 0, all trajectories converge to the single steady state solution. As *ρ*_1_+*ρ*_2_−1 goes from positive to negative, a Hopf bifurcation appears with oscillatory growth to infinity in a particular 2D plane (complex pair of roots with positive real part); convergence to this plane occurs along the third dimension (negative real root). For *ρ*_1_+*ρ*_2_−1 = 0, oscillations occur in two dimensions with a fixed radius dependent on the initial conditions (pair of purely imaginary roots, similar to the rotation network); convergence to this 2D plane of rotation occurs along the third dimension (negative real root).

It is worth emphasizing that the simple two-parameter condition *ρ*_1_+*ρ*_2_−1 > 0 and the corresponding stability phase space displayed in Fig. 6B were determined solely from consideration of the influence topology, which is itself completely defined by the graph of nodes and signed directed edges in Fig. 6A. Aside from the signs of the stoichiometries and monotonicities, no further specification of the exact functional forms of the reactions was required, nor was the number of steady states necessary to specify (only that they should all lie outside the unstable domain displayed in Fig. 6B for assurance of the network’s stability).

#### van der Pol network

The van der Pol network was first proposed in 1926 [25] as a model for stable oscillations in an electronic circuit:

$$\begin{array}{c} {\ddot{x}}_{1}-\mu \left(1-x_{1}^{2}\right){\dot{x}}_{1}+x_{1}=0. \end{array}$$ (71)

This second-order differential equation can be transformed to the following system of first-order differential equations through use of the Liénard transformation [35], $x_{2}=x_{1}-x_{1}^{3}/3-{\dot{x}}_{1}/\mu$, to yield:

$$\begin{array}{c} {\dot{x}}_{1}=\;\;\;k_{1}x_{1}\;\;\;-k_{2}x_{1}^{3}\;\;\;-k_{3}x_{2} \end{array}$$ (72)

$$\begin{array}{c} {\dot{x}}_{2}=\;\;\;\sigma_{1}k_{1}x_{1}.\;\;\;\;\;\; \end{array}$$ (73)

**Fig 7:**
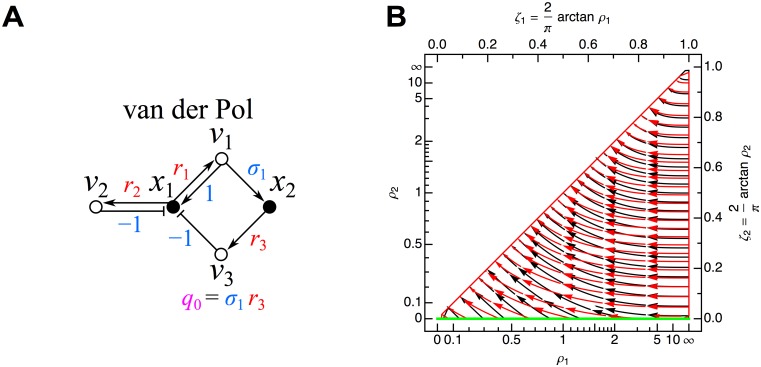
Van der Pol network. (A) Influence topology. Cycle compaction allows definition of *q*_0_ = *σ*_1_ *r*_3_. Temporal scaling of all Jacobian edges to |*q*_0_| leaves only *ρ*_1_ = *r*_1_/|*q*_0_| and *ρ*_2_ = *r*_2_/|*q*_0_|. (B) Stability phase space. Flows in the plot map the zones over which Δ_1_ (black) and Δ_2_ (red) are negative. The unstable zones for Δ_1_ (black) and Δ_2_ (red) completely overlap; in this region, two unstable eigenvalues obtain according to the Routh array. The green line indicates the possible set of solutions obtainable for the original van der Pol equations (Equations 72 and 73). See Fig. 6 for further details.

Its generalized form is:

$$\begin{array}{c} {\dot{x}}_{1}=\;\;\;V_{1}^{1}\;\;\;-V_{1}^{2}\;\;\;-V_{2}^{3} \end{array}$$ (74)

$$\begin{array}{c} {\dot{x}}_{2}=\;\;\;\sigma_{1}V_{1}^{1},\;\;\;\;\;\; \end{array}$$ (75)

corresponding to the influence topology displayed in Fig. 7A, with principal minors:

$$\begin{array}{ccc} b_{1} & = & \rho_{1}-\rho_{2} \end{array}$$ (76)

$$\begin{array}{ccc} b_{2} & = & \rho_{1}. \end{array}$$ (77)

For *b*_1_ = *c*_1_, both 1-cycles in the graph contribute. For $b_{2}=\bar{c_{1}c_{1}}-c_{2}$, only the 2-cycle (*r*_1_/|*q*_0_|)*σ*_1_(*r*_3_/|*q*_0_|)(−1) = −*ρ*_1_ contributes as the 1-cycles overlap with each other at the species node (similar overlap at a reaction node would also not be allowed). The Hurwitz determinants based on the expressions above for the principal minors are:

$$\begin{array}{ccc} \Delta_{1} & = & \rho_{2}-\rho_{1} \end{array}$$ (78)

$$\begin{array}{ccc} \Delta_{2} & = & \rho_{1}\left(\rho_{2}-\rho_{1}\right), \end{array}$$ (79)

corresponding to the stability phase space displayed in Fig. 7B. These can also be derived directly from the cycle-based definitions (where I have already removed terms that are clearly zero based on the influence topology in Fig. 7A):

$$\begin{array}{ccc} \Delta_{1} & = & -c_{1} \end{array}$$ (80)

$$\begin{array}{ccc} \Delta_{2} & = & c_{1}\cdot c_{2}. \end{array}$$ (81)

The corresponding stability phase space is displayed in Fig. 7B. For *ρ*_2_−*ρ*_1_ < 0, both Δ_1_ and Δ_2_ are negative, giving two unstable roots according to the number of sign changes in the Routh array *V*(+, −, +). Using the algebraic redefinition of Γ ≡ *ρ*_2_/*ρ*_1_ (Γ is strictly positive), the condition for instability becomes simply Γ < 1 (providing an example of a Hurwitz reduction).

For the original van der Pol network defined in Equations 72 and 73, the unique steady state solution is $x_{1}^{s}=x_{2}^{s}=0$ with *ρ*_1_ = *k*_1_/(*σ*_1_ *k*_3_) and *ρ*_2_ = 0 (this complete set of possible solutions is indicated by the green line in Fig. 7B). Since *ρ*_2_−*ρ*_1_ = −*k*_1_/(*σ*_1_ *k*_3_) < 0, this implies two unstable eigenvalues, which is consistent with the ever-present limit cycle in the network’s phase space.

Instead of the Liénard transformation, one could alternatively apply the more straightforward transformation of ${\dot{x}}_{1}=x_{2}$, which, upon a final swapping of *x*_1_ for *x*_2_, leads to:

$$\begin{array}{c} {\dot{x}}_{1}=\;\;\;k_{1}x_{1}\;\;\;-k_{2}x_{1}x_{2}^{2}\;\;\;-k_{3}x_{2} \end{array}$$ (82)

$$\begin{array}{c} {\dot{x}}_{2}=\;\;\;\sigma_{1}k_{1}x_{1}.\;\;\;\;\;\; \end{array}$$ (83)

These governing equations are identical to Equations 72 and 73 aside from the change of $k_{2}x_{1}^{3}\to k_{2}x_{1}x_{2}^{2}$ in the second reaction. The generalized form is:

$$\begin{array}{c} {\dot{x}}_{1}=\;\;\;V_{1}^{1}\;\;\;-V_{12}^{2}\;\;\;-V_{2}^{3} \end{array}$$ (84)

$$\begin{array}{c} {\dot{x}}_{2}=\;\;\;\sigma_{1}V_{1}^{1}.\;\;\;\;\;\; \end{array}$$ (85)

The influence topology is therefore identical to that shown in Fig. 7A aside from a single extra Jacobian arrow from *x*_2_ to *v*_2_. This extra connection, however, prevents the convenient parameter reduction obtained for the Liénard transformed network, with now three Jacobian parameters and one stoichiometric parameter required to specify the stability phase space instead of the two Jacobian parameters obtained above. This example demonstrates the still important aspect of the algebraic form for the governing equations. The specific algebraic structure taken for the governing equations can be critical in defining the influence topology, with the intriguing possibility—concretely demonstrated here for the van der Pol network—that transformations may exist to convert a given algebraically-defined network with complicated influence topology (requiring many parameters to specify its corresponding stability phase space) to a transformed version having a much simpler influence topology (lower dimensional stability phase space). In any case, as both algebraic forms for this network are equivalent, the projection of the solution set onto the lower or higher dimensional influence topologies following from these algebraic definitions must of course lead to the same dynamics.

#### Lotka-Volterra network

**Fig 8:**
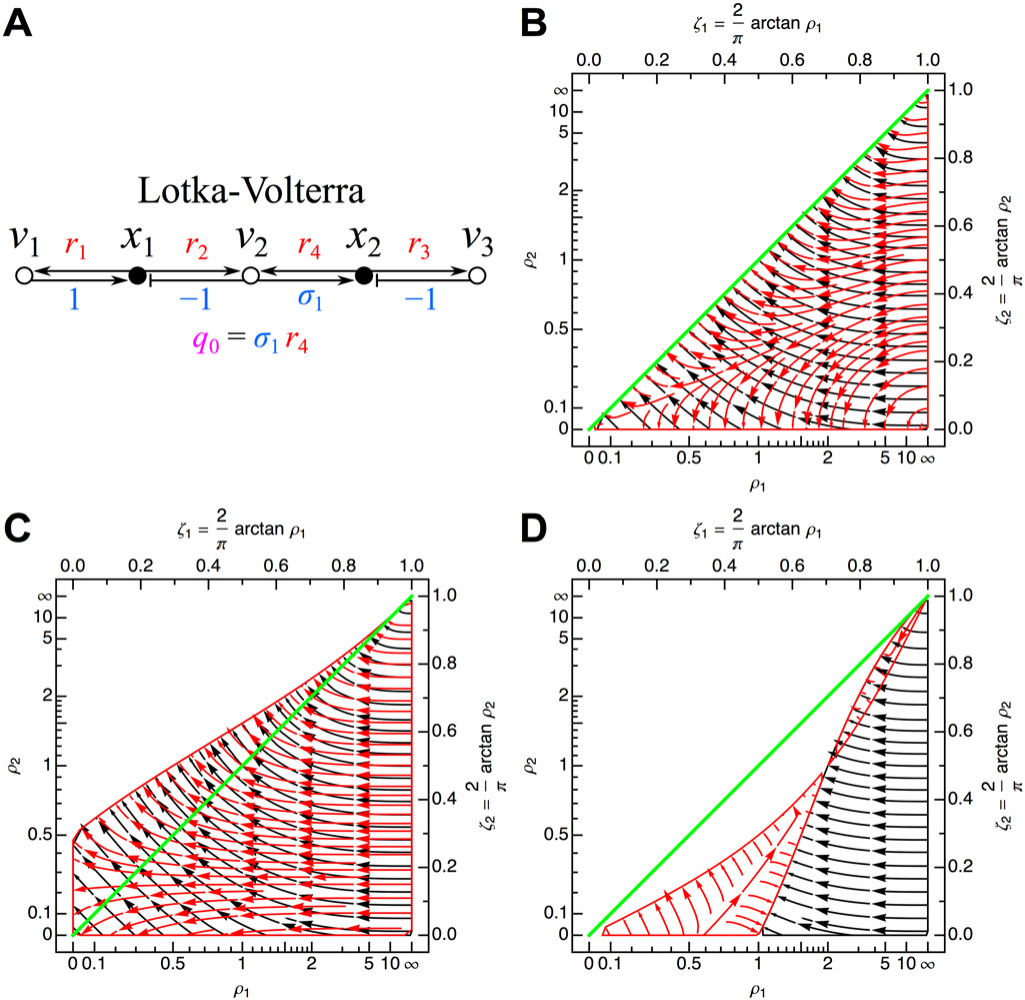
Lotka-Volterra network. (A) Influence topology. Cycle compaction allows definition of *q*_0_ = *σ*_1_ *r*_4_. Temporal scaling of all Jacobian edges to |*q*_0_| leaves only *ρ*_1_ = *r*_1_/|*q*_0_|, *ρ*_2_ = *r*_2_/|*q*_0_|, and *ρ*_3_ = *r*_3_/|*q*_0_|. The stability phase space is shown for (B) *ρ*_3_ = 1 (*σ*_1_ = 1), (C) *ρ*_3_ = 1/2 (*σ*_1_ = 2), and (D) *ρ*_3_ = 2 (*σ*_1_ = 1/2). Flows in the plot map the zones over which Δ_1_ (black) and Δ_2_ (red) are negative. See Fig. 6 for further details.

Lotka in 1920 [26], and independently Volterra in 1926 [27, 28], introduced the following network for modeling population oscillations:

$$\begin{array}{c} {\dot{x}}_{1}=\;\;\;k_{1}x_{1}\;\;\;-k_{2}x_{1}x_{2}\;\;\; \end{array}$$ (86)

$$\begin{array}{c} {\dot{x}}_{2}=\;\;\;\;\;\;\sigma_{1}k_{2}x_{1}x_{2}\;\;\;-k_{3}x_{2}, \end{array}$$ (87)

with *σ*_1_ = 1 typically assumed. The generalized Lotka-Volterra network is:

$$\begin{array}{c} {\dot{x}}_{1}=\;\;\;V_{1}^{1}\;\;\;-V_{12}^{2}\;\;\; \end{array}$$ (88)

$$\begin{array}{c} {\dot{x}}_{2}=\;\;\;\;\;\;\sigma_{1}V_{12}^{2}\;\;\;-V_{2}^{3}, \end{array}$$ (89)

corresponding to the influence topology shown in Fig. 8A, with principal minors (Equation 10):

$$\begin{array}{ccc} b_{1} & = & 1+\rho_{1}-\rho_{2}-\rho_{3} \end{array}$$ (90)

$$\begin{array}{ccc} b_{2} & = & \rho_{1}+\rho_{2}\rho_{3}-\rho_{1}\rho_{3}, \end{array}$$ (91)

and Hurwitz determinants:

$$\begin{array}{ccc} \Delta_{1} & = & \rho_{2}+\rho_{3}-\rho_{1}-1 \end{array}$$ (92)

$$\begin{array}{ccc} \Delta_{2} & = & \left(\rho_{2}+\rho_{3}-\rho_{1}-1\right)\left(\rho_{1}+\rho_{2}\rho_{3}-\rho_{1}\rho_{3}\right). \end{array}$$ (93)

The above results can also be obtained directly from the cycle product-based expressions of the Hurwitz determinants (Equations 17 and 18):

$$\begin{array}{ccc} \Delta_{1} & = & -c_{1} \end{array}$$ (94)

$$\begin{array}{ccc} \Delta_{2} & = & -c_{1}\cdot \bar{c_{1}c_{1}}, \end{array}$$ (95)

where I have already removed terms that are clearly zero based on the influence topology. The second term in the above product for Δ_2_ corresponds to pairs of non-overlapping 1-cycles ($\bar{c_{1}c_{1}}$) of which there are clearly three in the graph of the influence topology (yielding the second group of terms in Equation 93). The stability phase space over *ρ*_1_-*ρ*_2_ is displayed for different values of *ρ*_3_ in Figs. 8B-D.

For the more traditional functional form of the Lotka-Volterra system given in Equations 86 and 87, the unique steady state solution is:

$$\begin{array}{ccc} x_{1}^{s} & = & k_{3}/\left(\sigma_{1}k_{2}\right) \end{array}$$ (96)

$$\begin{array}{ccc} x_{2}^{s} & = & k_{1}/k_{2}. \end{array}$$ (97)

At the steady state, *r*_1_ = *k*_1_, *r*_2_ = *k*_1_, *r*_3_ = *k*_3_, *r*_4_ = *k*_3_, and *q*_0_ = *σ*_1_ *r*_4_ = *σ*_1_ *k*_3_, giving:

$$\begin{array}{ccc} \rho_{1} & = & \frac{1}{\sigma_{1}}\frac{k_{1}}{k_{3}} \end{array}$$ (98)

$$\begin{array}{ccc} \rho_{2} & = & \frac{1}{\sigma_{1}}\frac{k_{1}}{k_{3}} \end{array}$$ (99)

$$\begin{array}{ccc} \rho_{3} & = & \frac{1}{\sigma_{1}}. \end{array}$$ (100)

No matter the value of *σ*_1_, the steady-state solution will always lie on the *ρ*_1_-*ρ*_2_ diagonal in the stability phase space (green line in Figs. 8B-D). The Hurwitz determinants are:

$$\begin{array}{ccc} \Delta_{1} & = & \rho_{3}-1 \end{array}$$ (101)

$$\begin{array}{ccc} \Delta_{2} & = & \left(\rho_{3}-1\right)\rho_{1}. \end{array}$$ (102)

For the typically assumed value of *σ*_1_ = 1, we obtain *ρ*_3_ = 1 and *ρ*_1_ = *ρ*_2_ = *k*_1_/*k*_3_ and zero for both Routh-Hurwitz conditions: Δ_1_ = 0 and Δ_2_ = 0 (Fig. 8B), which prevents any conclusion about the stability of the network at first order. For *σ*_1_ = 2, *ρ*_3_ = 1/2 and *ρ*_1_ = *ρ*_2_ = *k*_1_/(2*k*_3_) with Δ_1_ = −1/2 and Δ_2_ = −*k*_1_/(4*k*_3_), giving two sign changes in the Routh array *V*(+, −, +) and therefore two eigenvalues with positive real parts (Fig. 8C), with the dynamical solutions corresponding to an oscillatory divergence to infinity. For *σ*_1_ = 1/2, *ρ*_3_ = 2 and *ρ*_1_ = *ρ*_2_ = 2*k*_1_/*k*_3_, with Δ_1_ = 1 and Δ_2_ = 2*k*_1_/*k*_3_ implying a stable network (Fig. 8D), characterized by an oscillatory convergence to the steady state solution.

As already stated above, the reaction functions defined in Equations 86 and 87 entail the restriction of the steady state solution to the *ρ*_1_-*ρ*_2_ diagonal in the stability phase space. The steady state solution can be shifted off the diagonal (even for *σ* = 1 in Fig. 8B) in a way that preserves the influence topology through addition of a constant reaction to one or both of the original governing equations (Equations 86 and 87), or through the introduction of more general reaction functions, for example:

$$\begin{array}{c} \;{\dot{x}}_{1}=\;\;\;k_{1}\exp x_{1}\;\;\;\;-k_{2}\sqrt{x_{1}}\arctan x_{2}\;\;\;\;\; \end{array}$$ (103)

$$\begin{array}{c} \;{\dot{x}}_{2}=\;\;\;\;\;\;\;\sigma_{1}k_{2}\sqrt{x_{1}}\arctan x_{2}\;\;\;\;-k_{3}\log(1+x_{2}),\; \end{array}$$ (104)

The assumption of other functional forms for the reactions might additionally allow for the existence of more than one steady state solution.

#### Brusselator network

**Fig 9:**
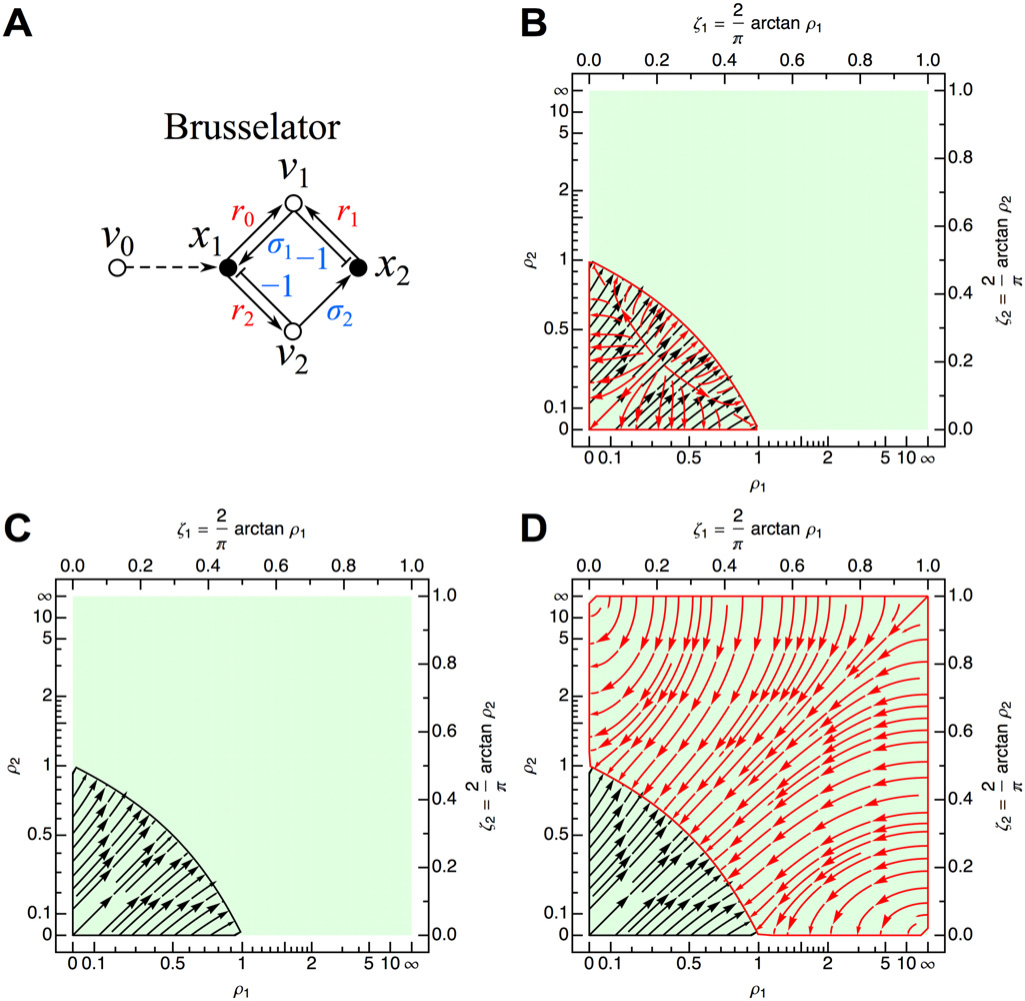
Brusselator network. (A) Influence topology. Temporal scaling of all Jacobian edges to |*r*_0_| gives *ρ*_1_ = *r*_1_/|*r*_0_| and *ρ*_2_ = *r*_2_/|*r*_0_|; *σ*_1_ and *σ*_2_ must be specified as well. The stability phase space is shown for *σ*_1_ = 1 and the following values for *σ*_2_: (B) *σ*_2_ = 1/2, (C) *σ*_2_ = 1, and (D) *σ*_2_ = 2. Flows in the plot map the zones over which Δ_1_ (black) and Δ_2_ (red) are negative. See Fig. 6 for further details.

The Brusselator was proposed by Prigogine & Lefever in 1968 [29] to account for oscillations in the Belousov-Zhabotinsky reaction [36, 37]:

$$\begin{array}{c} {\dot{x}}_{1}=\;\;\;k_{0}\;\;\;+\sigma_{1}k_{1}x_{1}^{2}x_{2}\;\;\;-k_{2}x_{1} \end{array}$$ (105)

$$\begin{array}{c} {\dot{x}}_{2}=\;\;\;\;\;\;-k_{1}x_{1}^{2}x_{2}\;\;\;+\sigma_{2}k_{2}x_{1}, \end{array}$$ (106)

with *σ*_1_ = 1 and *σ*_2_ < 1 (by definition of the original Brusselator network). Its generalized version is:

$$\begin{array}{c} {\dot{x}}_{1}=\;\;\;V^{0}\;\;\;+\sigma_{1}V_{12}^{1}\;\;\;-V_{1}^{2} \end{array}$$ (107)

$$\begin{array}{c} {\dot{x}}_{2}=\;\;\;\;\;\;-V_{12}^{1}\;\;\;+\sigma_{2}V_{1}^{2}, \end{array}$$ (108)

corresponding to the influence topology shown in Fig. 9A, with principal minors:

$$\begin{array}{ccc} b_{1} & = & \sigma_{1}-\rho_{1}-\rho_{2} \end{array}$$ (109)

$$\begin{array}{ccc} b_{2} & = & \rho_{1}\rho_{2}-\sigma_{1}\sigma_{2}\rho_{1}\rho_{2}, \end{array}$$ (110)

and Hurwitz determinants:

$$\begin{array}{ccc} \Delta_{1} & = & \rho_{1}+\rho_{2}-\sigma_{1} \end{array}$$ (111)

$$\begin{array}{ccc} \Delta_{2} & = & \left(\rho_{1}+\rho_{2}-\sigma_{1}\right)\rho_{1}\rho_{2}\left(1-\sigma_{1}\sigma_{2}\right). \end{array}$$ (112)

These expressions can also be obtained directly from the cycle-based defitions of Equations 17 and 18 (where I have already removed terms that are zero):

$$\begin{array}{ccc} \Delta_{1} & = & -c_{1} \end{array}$$ (113)

$$\begin{array}{ccc} \Delta_{2} & = & -c_{1}\cdot \bar{c_{1}c_{1}}+c_{1}\cdot c_{2}. \end{array}$$ (114)

The stability phase space is displayed in Figs. 9B-D for *σ*_1_ = 1 and different values of *σ*_2_. If 1−*σ*_1_ *σ*_2_ is positive, then instability can only occur for *ρ*_1_+*ρ*_2_−*σ*_1_ < 0 (Fig. 9B). Defining Ψ ≡ (*ρ*_1_+*ρ*_2_) (with Ψ strictly positive), this amounts to Ψ < *σ*_1_ for instability, or, upon the further algebraic redefintion (Hurwitz reduction) Γ ≡ Ψ/*σ*_1_ with Γ strictly positive, this becomes simply Γ < 1.

For the original Brusselator, *σ*_1_ = 1 and *σ*_2_ = (*k*_2_−*a*)/*k*_2_ (or 1−*σ*_2_ = *a*/*k*_2_). Both *σ*_2_ and *k*_2_ are assumed greater than zero, implying the further restrictions of *k*_2_ > *a* and 0 < *σ*_2_ < 1. These definitions give:

$$\begin{array}{ccc} \Delta_{1} & = & \rho_{1}+\rho_{2}-1 \end{array}$$ (115)

$$\begin{array}{ccc} \Delta_{2} & = & \left(\rho_{1}+\rho_{2}-1\right)\rho_{1}\rho_{2}\frac{a}{k_{2}}. \end{array}$$ (116)

For *ρ*_1_+*ρ*_2_−1 < 0, both Δ_1_ and Δ_2_ are simultaneously negative, corresponding to a transition from zero to two unstable eigenvalues and therefore the possibility of a Hopf bifurcation and limit cycle. The single steady state for the Brusselator is located at:

$$\begin{array}{ccc} x_{1}^{s} & = & k_{0} \end{array}$$ (117)

$$\begin{array}{ccc} x_{2}^{s} & = & \frac{k_{2}-a}{k_{0}k_{1}}, \end{array}$$ (118)

giving

$$\begin{array}{ccc} \rho_{1} & = & \frac{k_{0}^{2}k_{1}}{2(k_{2}-a)} \end{array}$$ (119)

$$\begin{array}{ccc} \rho_{2} & = & \frac{k_{2}}{2(k_{2}-a)}. \end{array}$$ (120)

Upon appropriate choices for *a*, *k*_0_, *k*_1_, and *k*_2_, both *ρ*_1_ and *ρ*_2_ can assume any value in the stability phase space (green region of Figs. 9B-D). The condition for instability to obtain, *ρ*_1_+*ρ*_2_−1 < 0, becomes:

$$\begin{array}{c} \frac{k_{0}^{2}k_{1}}{a}-\frac{k_{2}}{a}+2<0. \end{array}$$ (121)

Defining $A^{2}\equiv k_{0}^{2}k_{1}/a$ and *B* ≡ *k*_2_/*a*−1, this yields the standard result of *B* > 1+*A* ^2^.

For 1−*σ*_1_ *σ*_2_ = 0, Δ_2_ = 0, implying a reduction in dimensionality and therefore only a single real eigenvalue with sign opposite to that of Δ_1_ (Fig. 9C). For 1−*σ*_1_ *σ*_2_ < 0, sign(Δ_2_) = −sign(Δ_1_), which for Δ_1_ ≠ 0 will always generate one stable and one unstable eigenvalue according to the one sign change in the Routh array (either *V*(+, −, −) or *V*(+, +, −)) (Fig. 9D).

#### Sel’kov network

Sel’kov in 1968 [30] proposed the following simple model to account for glycolytic oscillations:

$$\begin{array}{c} {\dot{x}}_{1}=\;\;\;\;\;\;-k_{1}x_{1}\;\;\;+\sigma_{1}k_{2}x_{1}^{2}x_{2}\;\;\;+\sigma_{2}k_{3}x_{2} \end{array}$$ (122)

$$\begin{array}{c} {\dot{x}}_{2}=\;\;\;k_{0}\;\;\;\;\;\;-k_{2}x_{1}^{2}x_{2}\;\;\;-k_{3}x_{2}, \end{array}$$ (123)

with *σ*_1_ and *σ*_2_ equal to 1. The generalized version is:

$$\begin{array}{c} {\dot{x}}_{1}=\;\;\;\;\;\;-V_{1}^{1}\;\;\;+\sigma_{1}V_{12}^{2}\;\;\;+\sigma_{2}V_{2}^{3} \end{array}$$ (124)

$$\begin{array}{c} {\dot{x}}_{2}=\;\;\;V^{0}\;\;\;\;\;\;-V_{12}^{2}\;\;\;-V_{2}^{3}. \end{array}$$ (125)

**Fig 10:**
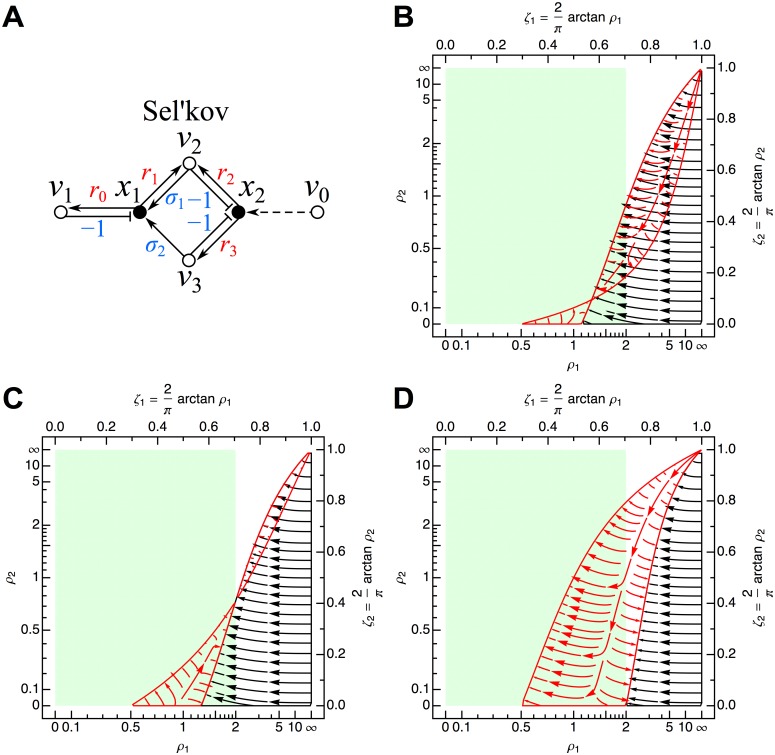
Sel’kov network. (A) Influence topology. Temporal scaling of all Jacobian edges to |*r*_0_| gives the parameters *ρ*_1_ = *r*_1_/|*r*_0_|, *ρ*_2_ = *r*_2_/|*r*_0_|, and *ρ*_3_ = *r*_3_/|*r*_0_|. The positive stoichiometric terms *σ*_1_ and *σ*_2_ must also be specified independently. The stability phase space is shown for *σ*_1_ = *σ*_2_ = 1 and (B) *ρ*_3_ = 1/10; (C) *ρ*_3_ = 1/4; and (D) *ρ*_3_ = 1. Flows in the plot map the zones over which Δ_1_ (black) and Δ_2_ (red) are negative. See Fig. 6 for further details.

The influence topology is shown in Fig. 10A, with principal minors:

$$\begin{array}{ccc} b_{1} & = & -1+\sigma_{1}\rho_{1}-\rho_{2}-\rho_{3} \end{array}$$ (126)

$$\begin{array}{ccc} b_{2} & = & \rho_{2}+\rho_{3}-\sigma_{1}\rho_{1}\rho_{3}-\sigma_{2}\rho_{1}\rho_{3}, \end{array}$$ (127)

and Hurwitz determinants:

$$\begin{array}{ccc} \Delta_{1} & = & 1+\rho_{2}+\rho_{3}-\sigma_{1}\rho_{1} \end{array}$$ (128)

$$\begin{array}{ccc} \Delta_{2} & = & \left(1+\rho_{2}+\rho_{3}-\sigma_{1}\rho_{1}\right)\left(\rho_{2}+\rho_{3}-\sigma_{1}\rho_{1}\rho_{3}-\sigma_{2}\rho_{1}\rho_{3}\right). \end{array}$$ (129)

This result can also be obtained directly from the cycle-based definitions (where I have already removed terms that are zero):

$$\begin{array}{ccc} \Delta_{1} & = & -c_{1} \end{array}$$ (130)

$$\begin{array}{ccc} \Delta_{2} & = & -c_{1}\cdot \bar{c_{1}c_{1}}+c_{1}\cdot c_{2}. \end{array}$$ (131)

The corresponding stability phase space is displayed in Figs. 10B-D for *σ*_1_ = *σ*_2_ = 1 and different values of *ρ*_3_.

For the original network (Equations 122 and 123) with *σ*_1_ = *σ*_2_ = 1, the Hurwitz determinants simplify to:

$$\begin{array}{ccc} \Delta_{1} & = & 1+\rho_{2}+\rho_{3}-\rho_{1} \end{array}$$ (132)

$$\begin{array}{ccc} \Delta_{2} & = & \left(1+\rho_{2}+\rho_{3}-\rho_{1}\right)\left(\rho_{2}+\rho_{3}-2\rho_{1}\rho_{3}\right). \end{array}$$ (133)

The steady state solution is:

$$\begin{array}{ccc} x_{1}^{s} & = & \frac{k_{0}}{k_{1}} \end{array}$$ (134)

$$\begin{array}{ccc} x_{2}^{s} & = & {\left(\frac{k_{0}k_{2}}{k_{1}^{2}}+\frac{k_{3}}{k_{0}}\right)}^{-1}, \end{array}$$ (135)

at which

$$\begin{array}{ccc} \rho_{1} & = & 2{\left(1+\frac{k_{1}^{2}k_{3}}{k_{0}^{2}k_{2}}\right)}^{-1} \end{array}$$ (136)

$$\begin{array}{ccc} \rho_{2} & = & \frac{k_{0}^{2}k_{2}}{k_{1}^{3}} \end{array}$$ (137)

$$\begin{array}{ccc} \rho_{3} & = & \frac{k_{3}}{k_{1}}. \end{array}$$ (138)

The Sel’kov stability phase space shows interesting structure for three different values of *ρ*_3_. A Hopf bifurcation and limit cycle are only possible if *ρ*_3_ < 1/4 and *ρ*_1_ and *ρ*_2_ map the network to the zone in which both Δ_1_ and Δ_2_ are less than zero (small black/red overlap region in Fig. 10B).

#### Repressilator network

**Fig 11:**
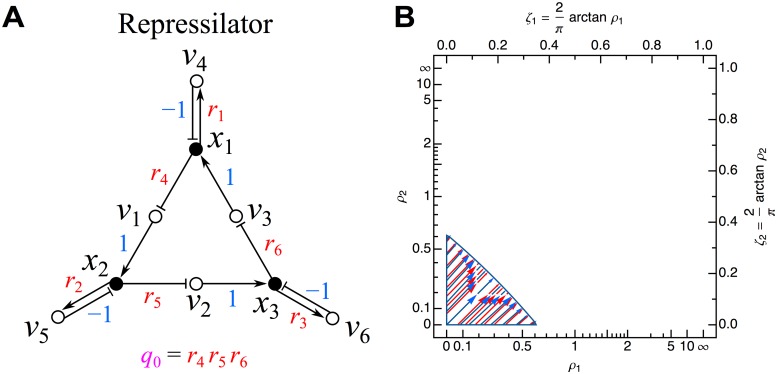
Repressilator network. (A) Influence topology. Cycle compaction leads to definition of *q*_0_ = *r*_4_ *r*_5_ *r*_6_, which is further removed after temporal scaling, leaving only *ρ*_1_ = *r*_1_/|*q*_0_|^1/3^, *ρ*_2_ = *r*_2_/|*q*_0_|^1/3^, and *ρ*_3_ = *r*_3_/|*q*_0_|^1/3^. (B) Stability phase space for *ρ*_3_ = 1. Flows in the plot map the zones over which Δ_1_ (black), Δ_2_ (red), and Δ_3_ (blue) are negative. For *ρ*_3_ < 1, the domain of instability will be increased towards the upper right (and oppositely for larger *ρ*_3_). No green zone is indicated here due to the dependence of the exact steady state solution(s) on the choice of Hill coefficients in the definition of the original network in Equations 139–141. See Fig. 6 for further details.

Several different genetic oscillators have been investigated since the first proposal of Monod and Jacob in 1961 [38]. One particularly famous example is the Repressilator [31–34]:

$$\begin{array}{c} \;{\dot{x}}_{1}=\;\;\;\;\;\;\;\;\;\;k_{3}\frac{1}{1+x_{3}^{c}}\;\;\;\;-k_{4}x_{1}\;\;\;\;\;\; \end{array}$$ (139)

$$\begin{array}{c} \;{\dot{x}}_{2}=\;\;\;\;k_{1}\frac{1}{1+x_{1}^{a}}\;\;\;\;\;\;\;\;\;\;\;\;\;-k_{5}x_{2}\;\;\; \end{array}$$ (140)

$$\begin{array}{c} \;{\dot{x}}_{3}=\;\;\;\;\;\;\;k_{2}\frac{1}{1+x_{2}^{b}}\;\;\;\;\;\;\;\;\;\;\;\;\;-k_{6}x_{3}, \end{array}$$ (141)

which has the following generalized form (Fig. 11A):

$$\begin{array}{c} {\dot{x}}_{1}=\;\;\;\;\;\;\;\;\;V_{\bar{3}}^{3}\;\;\;-V_{1}^{4}\;\;\;\;\;\; \end{array}$$ (142)

$$\begin{array}{c} {\dot{x}}_{2}=\;\;\;V_{\bar{1}}^{1}\;\;\;\;\;\;\;\;\;\;\;\;-V_{2}^{5}\;\;\; \end{array}$$ (143)

$$\begin{array}{c} {\dot{x}}_{3}=\;\;\;\;\;\;V_{\bar{2}}^{2}\;\;\;\;\;\;\;\;\;\;\;\;-V_{3}^{6}, \end{array}$$ (144)

with principal minors:

$$\begin{array}{ccc} b_{1} & = & -\rho_{1}-\rho_{2}-\rho_{3} \end{array}$$ (145)

$$\begin{array}{ccc} b_{2} & = & \rho_{1}\rho_{2}+\rho_{1}\rho_{3}+\rho_{2}\rho_{3} \end{array}$$ (146)

$$\begin{array}{ccc} b_{3} & = & -\rho_{1}\rho_{2}\rho_{3}-1, \end{array}$$ (147)

and Hurwitz determinants:

$$\begin{array}{ccc} \Delta_{1} & = & \rho_{1}+\rho_{2}+\rho_{3} \end{array}$$ (148)

$$\begin{array}{ccc} \Delta_{2} & = & \left(\rho_{1}+\rho_{2}+\rho_{3}\right)\left(\rho_{1}\rho_{2}+\rho_{1}\rho_{3}+\rho_{2}\rho_{3}\right)-\rho_{1}\rho_{2}\rho_{3}-1 \end{array}$$ (149)

$$\begin{array}{ccc} \Delta_{3} & = & \left(\rho_{1}\rho_{2}\rho_{3}+1\right)\Delta_{2}, \end{array}$$ (150)

corresponding to the stability phase space displayed in Fig. 11B (for *ρ*_3_ = 1). These expressions can also be obtained directly from the cycle-based forms (where I have already removed terms that are clearly zero based on the influence topology):

$$\begin{array}{ccc} \Delta_{1} & = & -c_{1} \end{array}$$ (151)

$$\begin{array}{ccc} \Delta_{2} & = & -c_{1}\cdot \bar{c_{1}c_{1}}+c_{0}\cdot \bar{c_{1}c_{1}c_{1}}+c_{0}\cdot c_{3}. \end{array}$$ (152)

$$\begin{array}{ccc} \Delta_{3} & = & c_{1}\cdot \bar{c_{1}c_{1}}\cdot \bar{c_{1}c_{1}c_{1}}-c_{0}\cdot \bar{c_{1}c_{1}c_{1}}\cdot \bar{c_{1}c_{1}c_{1}}\\ & & +\;c_{1}\cdot \bar{c_{1}c_{1}}\cdot c_{3}-2c_{0}\cdot \bar{c_{1}c_{1}c_{1}}\cdot c_{3}\\ & & -\;c_{0}\cdot c_{3}\cdot c_{3}. \end{array}$$ (153)

After some cancellation in Δ_2_, instability can be shown to arise for:

$$\begin{array}{c} \rho_{1}^{2}\rho_{2}+\rho_{1}^{2}\rho_{3}+\rho_{2}^{2}\rho_{1}+\rho_{2}^{2}\rho_{3}+\rho_{3}^{2}\rho_{1}+\rho_{3}^{2}\rho_{2}+2\rho_{1}\rho_{2}\rho_{3}<1. \end{array}$$ (154)

The transition from positive to negative occurs simulatenously for Δ_2_ and Δ_3_, implying the simultaneous appearance of two unstable eigenvalues according to the number of sign changes in the Routh array *V*(+, +, −, +) (necessary condition for a Hopf bifurcation). The purely positive sum of terms on the left-hand side is symmetric with respect to exchange of the *ρ*_*i*_ (exchanging the *r*_*i*_ in the influence topology has no effect on the cycle definitions or their non-overlapping contributions to the principal minors). Defining Ψ as this left-hand-side quantity (topological reduction) with Ψ strictly positive amounts to the single parameter condition of Ψ < 1 for instability.

As the number and positions of the steady state solutions depend sensitively on the Hill coefficients (yielding complicated expressions even for *a* = *b* = *c* = 1), no simple general expression exists. Whether the complete stability phase space or only a portion is accessible for a given choice of *a*, *b*, and *c* may not have a simple answer.

If the 3-cycle is positive rather than negative, the Hurwitz determinants are then:

$$\begin{array}{ccc} \Delta_{1} & = & \rho_{1}+\rho_{2}+\rho_{3} \end{array}$$ (155)

$$\begin{array}{ccc} \Delta_{2} & = & \left(\rho_{1}+\rho_{2}+\rho_{3}\right)\left(\rho_{1}\rho_{2}+\rho_{1}\rho_{3}+\rho_{2}\rho_{3}\right)-\rho_{1}\rho_{2}\rho_{3}+1 \end{array}$$ (156)

$$\begin{array}{ccc} \Delta_{3} & = & \left(\rho_{1}\rho_{2}\rho_{3}-1\right)\Delta_{2}. \end{array}$$ (157)

The only possibility for instability is now through Δ_3_, which will be negative if Ψ ≡ *ρ*_1_ *ρ*_2_ *ρ*_3_ < 1. In this region of instability, the Routh array is *V*(+, +, +, −), implying only one unstable eigenvalue. It is important to note that while there is no possibility for a Hopf bifurcation to arise at any steady state solution, this does not by itself rule out the possibility of a limit cycle.

### 6 Analysis of General Networks

In the above I have treated several examples of networks consisting of a single level of overlapping cycles (in graph theory, “strongly connected components”) and, in some cases, an additional constant upstream reaction node (orphan reaction). In this section, I show how this approach can be generalized to serve as a useful starting point for the analysis of more general networks through upstream/downstream network partitioning. The basic algorithm, illustrated in the following examples, is simple and intuitive. A given network can be partitioned into its upstream/downstream levels, with each level consisting of overlapping cycles. Analysis of the network’s stability through its influence topology then proceeds on a level-by-level basis starting from the most upstream level and then proceeding further downstream.

**Fig 12:**
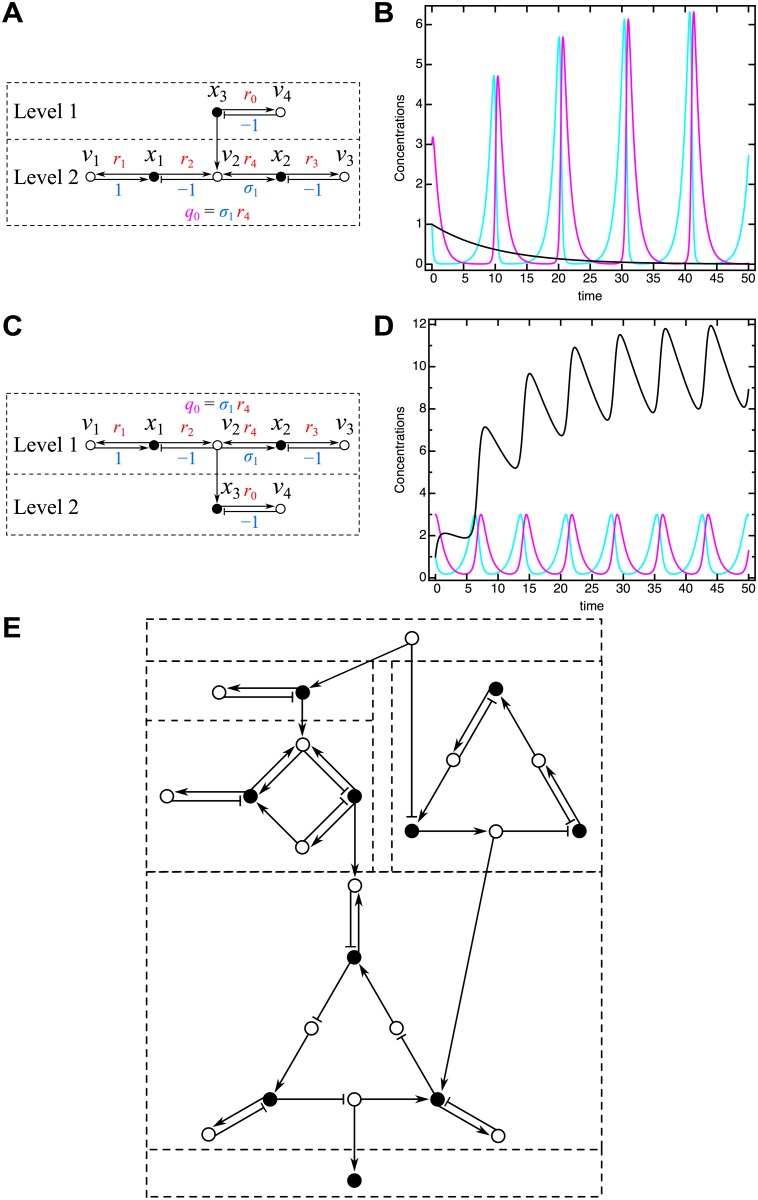
Analysis of general networks. (A) Network consisting of two distinct levels of overlapping cycles. (B) Species trajectories (*χ*_1_, cyan; *χ*_2_, magenta; *χ*_3_, black) of an explicit algebraic version (Equations 158–160) of a network having the influence topology shown in A (see §6 for details). (C) Similar two-level network as in A, but with the levels swapped. (D) Species trajectories (*χ*_1_, cyan; *χ*_2_, magenta; *χ*_3_, black) of an explicit algebraic version (Equations 161–163) of a network having the influence topology shown in C (see §6 for details). (E) Example of a more complicated multilevel network.

Consider the simple example shown in Fig. 12A of a network consisting of two such levels. A specific algebraic form for this network is:

$$\begin{array}{c} \;\;\;V_{1}^{1}\;\;\;V_{123}^{2}\;\;\;V_{2}^{3}\;\;\;V_{3}^{4} \end{array}$$

$$\begin{array}{c} {\dot{x}}_{1}=\;\;\;x_{1}\;\;\;-\left(1+x_{3}\right)x_{1}x_{2}\;\;\;\;\;\; \end{array}$$ (158)

$$\begin{array}{c} {\dot{x}}_{2}=\;\;\;\;\;\;\left(1+x_{3}\right)x_{1}x_{2}\;\;\;-x_{2},\;\;\; \end{array}$$ (159)

$$\begin{array}{c} {\dot{x}}_{3}=\;\;\;\;\;\;\;\;\;\;\;\;-0.1x_{3}. \end{array}$$ (160)

Trajectories of the species in this network for the specific choice of initial conditions of *x*_1_(0) = 1, *x*_2_(0) = 3, and *x*_3_(0) = 1 is shown in Fig. 12B. Here, *x*_1_ and *x*_2_ exhibit Lotka-Volterra-like oscillations that grow in an asymptotic fashion towards a fixed amplitude, with *x*_3_ decaying to 0. Starting from Level 1, the 1-cycle is negative and therefore stable (see §4), with *x*_3_ in the vicinity of the steady state asymptotically approaching a steady value (in this case, to the value of 0, but any other fixed value would produce the same result). Species *x*_3_ then serves effectively as a constant species input to Level 2, in the same manner as an orphan species (see §3), meaning we can then proceed to consideration of Level 2. In the vicinity of the steady state, Level 2 can be considered as an (asymptotically) autonomous subnetwork. This level has an influence topology identical to the Lotka-Volterra network, permitting the possibility for instability.

Now consider a network similar to that shown in Fig. 12A but with the levels interchanged (Fig. 12C). An explicit algebraic representation of such a network is:

$$\begin{array}{c} \;\;\;V_{1}^{1}\;\;\;V_{12}^{2}\;\;\;V_{2}^{3}\;\;\;V_{3}^{4} \end{array}$$

$$\begin{array}{c} {\dot{x}}_{1}=\;\;\;x_{1}\;\;\;-x_{1}x_{2}\;\;\;\;\;\; \end{array}$$ (161)

$$\begin{array}{c} {\dot{x}}_{2}=\;\;\;\;\;\;x_{1}x_{2}\;\;\;-x_{2},\;\;\; \end{array}$$ (162)

$$\begin{array}{c} {\dot{x}}_{3}=\;\;\;\;\;\;x_{1}x_{2}\;\;\;\;\;\;-0.1x_{3}. \end{array}$$ (163)

Oscillations can now occur at the Lotka-Volterra-like Level 1. In the presence of such an oscillatory input from above, Level 2 is no longer an (asymptotically) *autonomous* subnetwork (the approach presented in this manuscript is only valid for autonomous systems). For the initial conditions of *x*_1_(0) = 1, *x*_2_(0) = 3, and *x*_3_(0) = 1, oscillations generated in Level 1 drive oscillations at Level 2 (oscillatory *x*_3_) as shown in Fig. 12D.

Analysis of more general networks like that shown in Fig. 12E should again proceed from upstream to downstream with separate consideration of distinct sets of overlapping cycles that may appear in parallel (within the same level).

### 7 Defining the Fundamental Set of Influence Topologies

While studying the set of all possible interaction networks (dynamical systems) makes little sense, the set of all possible influence topologies is denumerable and can therefore be systematically studied. Based on the above level-by-level analysis, we can restrict our attention to a *fundamental* set of influence topologies corresponding to all possible graphs of overlapping cycles. How to actually go about algorithmically enumerating all possible signed directed bipartite graphs comprised purely of overlapping cycles for a fixed number of *n* species and *m* reactions while avoiding repeats presents a significant challenge. Aside from this issue, even a small number of species and reactions will generate a lengthy list due to the 2^*J*+*S*^ different possible unique sign assignments for the *J* Jacobian and *S* stoichiometric edges for a given directed bipartite graph.

The following node-based sign degeneracy significantly reduces the list of truly *unique* fundamental influence topologies. Consider a single influence topology graph displaying the positive/negative connections between the species *x*_1_, …, *x*_*n*_ and the reactions *v*_1_, …, *v*_*m*_. For the species node *x*_1_, we can make the variable substitution *y*_1_ = −*x*_1_. That this leads to negation of all its associated edges in the influence topology is simple to show. All Jacobian arrows emanating from node *y*_1_ are transformed to ∂*v*_*k*_/∂*y*_1_ = −∂*v*_*k*_/∂*x*_1_; all stoichiometric arrows pointing to node *y*_1_ are also negated as the governing equation for species 1 is now ${\dot{y}}_{1}=-{\dot{x}}_{1}=-f_{1}=-\sum_{k}v_{k}s_{1}^{k}=\sum_{k}v_{k}(-s_{1}^{k})$. Now consider the reaction node substitution *w*_1_ = −*v*_1_. All Jacobian arrows that point to this reaction are negated, ∂*w*_1_/∂*x*_*i*_ = −∂*v*_1_/∂*x*_*i*_; all stoichiometric arrows that emanate from this reaction will also be negated, as one can see from the governing equations, ${\dot{x}}_{i}=f_{i}=w_{1}s_{i}^{1}+\sum_{k\neq 1}v_{k}s_{i}^{k}=v_{1}(-s_{i}^{1})+\sum_{k\neq 1}v_{k}s_{i}^{k}$. Importantly, negation of either a species or reaction node will not change the sign of the cycles that include this node (nor, or course, the other cycles in the network), implying no change in the cycle-based Hurwitz determinants. The presence of this sign degeneracy significantly reduces the list of all possible unique influence topologies. A trivial example of this sign degeneracy is in the above analysis of *n*-cycle networks (see §4), for which only overall positive or negative *n*-cycles are necessary to consider: It is easy to show that the directed edges of any *n*-cycle network can be transformed by sign substitutions into either all arrows (*c*_*n*_ = 1) or a single blunt arrow and the rest arrows (*c*_*n*_ = −1).

Another important sign degeneracy that may further reduce the list of fundamental influence topologies arises within cycle compaction terms. Cycle compaction terms are either positive or negative due to the assumed fixed signs of the edges that comprise a given term. Here, it is more fundamental to consider the cycle compaction terms as defined by *all* of the edges that contribute to the overlap, not just those that contribute an unspecified parameter (as was the convention in Fig. 3). It is clear that any swapping of the sign of an even number of the edges that define a particular compaction term will not affect its sign. Sign degeneracy within cycle compaction terms is clearly related to the node-based sign degeneracy described above (for the case of *n*-cycle networks, this relationship is obvious), but the more general nature of this connection appears nontrivial.

Finally, influence topologies with seemingly distinct architectures might nevertheless have a similar set of cycle-based intersections (returning again to the idea of cycle compaction) and therefore the same implications for the Routh-Hurwitz conditions, suggesting the additional presence of architectural degeneracies. For example, imagine graphically swapping the fragment defined by parameters *eflm* with that defined by *gh* in Fig. 3B; such a change will have no impact on the overlapping cycles of the influence topology (the fundamental variables for stability analysis) or the associated stability phase space.

Proper accounting for these degeneracies would clearly simplify the general problem of connecting network topology with stability.

## Discussion

I have shown that examination of a network’s influence topology, which is based only on the signs of the stoichiometries and monotonicities of its reactions, already restricts the spectrum of its dynamical solutions (i.e. the precise numbers of unstable eigenvalues possible at each steady state) without having to determine the exact steady state solution(s). The influence topology of a network acts like a skeleton with bones set into joints that already delimit a potential range of movement; additional algebraic specification of the network acts like the tendons/ligaments/muscles/tissue that further restrict this range, in some cases still permitting sampling of the entire range of movement (e.g. the Jenkin-Maxwell and Brusselator networks) but in other cases significantly resticting this range (e.g. the van der Pol and Lotka-Volterra networks). The most striking aspect of the above hierarchical treatment (topology-then-algebra) is the dramatic reduction in parameters that is often possible, with the many different reaction constants that define the original algebraic network reduced to only one or a few topological parameters for analysis of its stability. For example, the nine reaction parameters plus three initial conditions that define the Jenkin-Maxwell network were immediately reduced to a two-parameter stability condition that could be further reduced to a single-parameter condition due to a topological symmetry. It is important to emphasize the solely topological basis of this parameter reduction.

The influence topology should be especially useful for systems biology and synthetic biology, where detailed information about biological reaction functions beyond the signs of their stoichiometries and monotonicities is often unavailable (e.g. unknown Hill coefficient [39], complicated transcriptional promoter regulation) or sometimes interesting to ignore (e.g. for robustness studies [40, 41]). The principal benefit of the influence topology is the readily accessible constraints it provides on a given network’s possible dynamics, revealing the potential to shift a steady state from stability to instability (or vice versa) as well as what types of bifurcation may be present (e.g. it provides necessary and fairly stringent conditions for a Hopf bifurcation). From a synthetic biology perspective, the influence topology provides an informative basis for better undestanding how the stability of a particular network could be changed (e.g., by changing a particular reaction’s cooperativity and therefore the steepness of a given reaction Jacobian, shifting the location of the steady state within the stability phase space).

Stoichiometry has been heavily emphasized in the past, almost always under the additional assumption of mass action kinetics [7, 42–44]. While stoichiometry can indeed reduce the dimensionality of the original set of ODEs that underlie the network’s dynamics and can play an important role in probing the possible number of steady states (multistationarity) of certain classes of networks, stoichiometry alone provides only a limited perspective on the general problem of network stability. As the above investigations of the influence topology show, the notions of stoichiometric scaling and especially cycle compaction prove that variables other than the individual stoichiometric terms are more suitable for examining a given network’s local steady-state stability. In particular, upon cycle compaction, multiple stoichiometric terms often end up being degenerate with themselves or, even more interestingly, with co-compacted Jacobian terms (see Fig. 3 as well as the analysis of the Jenkin-Maxwell, van der Pol, and Lotka-Volterra influence topologies).

Approaches for determining the number of steady states (multistationarity) of networks have also traditionally received more attention in the past than methods for testing steady state stability. It should be noted that the six classical networks considered above have (or can have, in the case of the Repressilator) only a single steady state solution no matter the values of the parameters that define the network. Whether this single steady state can become unstable and exactly how it becomes unstable (e.g. through a Hopf bifurcation) is then the interesting question, not steady state multiplicity. It should nevertheless be noted that for more general networks that share the same influence topology as the networks considered above (e.g. Sel’kov-*like* networks), multiple steady state solutions may be possible depending upon the exact form that the reaction functions take, but all of these steady states would still have to lie somewhere on the (unchanged) stability phase space defined by the influence topology. For an in depth analysis of multistationarity using the influence topology see [1, 2].

Important open questions are enumerated below.

**Open Question #1:** For each position in the stability phase space of a given influence topology, is it always possible to find an explicit algebraic definition of the network that would map at least one of the network’s steady states to this position? I have shown above that the algebraic definitions of several classical networks project either onto the entire stability phase space or only a part of it. The above question approaches this projection from the opposite viewpoint of the influence topology. A better understanding of this question would clearly be beneficial for the engineering of networks with desired properties (without requiring alteration of the network’s influence topology).

**Open Question #2:** What is the most appropriate definition for the *minimal* influence topology of a particular algebraically-defined network? In attempting to write down *the* influence topology for the van der Pol network above, we discovered that it depends on the particular algebraic expression of its governing equations. In this case, two different algebraic versions (the Liénard-transformed version and a canonical version) led to influence topologies that differed by only a single link, with the simpler network (Liénard-transformed) entailing significantly fewer dimensions to describe its complete stability phase space. This example immediately suggests the notion of a *minimal* influence topology for a given algebraic network (reachable by a suitable algebraic transformation) with an associated stability phase space having the fewest possible dimensions. Such a definition should account for possible degeneracy of the minimal topology; lowest dimensionality is likely insufficient to uniquely identify the most *minimal* topology. Potential degeneracies could at least be partially accounted for by adoption of further criteria, such as the influence topology with the smallest cycles, least number of cycles, and/or the least number of each type of edges. Algorithms that allow one to find the unique minimal influence topology (or degenerate set of minimal influence topologies) for a given algebraic network would be of great interest.

**Open Question #3:** What is the most appropriate definition for the fundamental set of influence topologies? Based on the denumerability of the influence topology, the notion of a fundamental set of all possible influence topologies was presented (§7). As argued above, this fundamental set should consist of influence topologies consisting of only overlapping cycles and must furthermore take into account the two forms of sign degeneracy identified above (arising at each node or within each compaction parameter) and any possible architectural degeneracies. Enumeration and examination of all non-degenerate fundamental influence topologies and their associated stability phase spaces for low dimensional networks (or low dimensional influence topologies) should significantly deepen our understanding of the connection between network topology and stability.

**Open Question #4:** Is it possible to *derive* the Routh-Hurwitz conditions from a purely topological perspective? In the above, I have shown that it is possible to *interpret* the Routh-Hurwitz conditions topologically, but to derive these conditions topologically is a much deeper challenge. The topological expressions of the Routh-Hurwitz conditions presented above are, as already pointed out, not “fully reduced” (see §1). Further reduction of these expressions will require development of a more explicit topological notation capable of accounting for the many different types of intersection that can potentially take place among the bipartite cycles of a network’s influence topology. As the notion of cycle compaction introduced above is based on cycle overlaps (see Fig. 3), it may offer a useful perspective on this problem.
