# Correction: Implications of Network Topology on Stability

**DOI:** 10.1371/journal.pone.0131347

**Published:** 2015-06-18

**Authors:** Ali Kinkhabwala

There is an error in Equation 34 within the subsection “Parameter Reduction of the Stability Phase Space” in the Results section. The absolute value notation is incorrect. Please view the complete, correct equation here:

$$\sigma_{j}^{k}\;=\;\frac{1}{\alpha_{k}}s_{j}^{k}$$

## References

[1] Kinkhabwala A (2015) Implications of Network Topology on Stability. PLoS ONE 10(3): e0122150 doi: 10.1371/journal.pone.0122150 25826219 10.1371/journal.pone.0122150 PMC4380337

